# Dynamically tunable long-range coupling enabled by bound state in the continuum

**DOI:** 10.1038/s41377-025-01975-y

**Published:** 2025-08-18

**Authors:** Haijun Tang, Can Huang, Yuhan Wang, Xiong Jiang, Ruiheng Jin, Yue Cui, Shumin Xiao, Qinghai Song

**Affiliations:** 1 Ministry of Industry and Information Technology Key Lab of Micro-Nano Optoelectronic Information System, Guangdong Provincial Key Laboratory of Semiconductor Optoelectronic Materials and Intelligent Photonic Systems, Harbin Institute of Technology, Shenzhen, China; 2 Pengcheng Laboratory, Shenzhen, China; 3 National Key Laboratory of Science and Technology on Advanced Composites in Special Environments, Harbin Institute of Technology, Harbin, China; 4 Quantum Science Center of Guangdong-Hongkong Macao Greater Bay Area, Shenzhen, China; 5 Heilongjiang Provincial Key Laboratory of Advanced Quantum Functional Materials and Sensor devices, Harbin Institute of Technology, Harbin, China; 6 Collaborative Innovation Center of Extreme Optics, Shanxi University, Taiyuan, Shanxi China

**Keywords:** Nanophotonics and plasmonics, Lasers, LEDs and light sources

## Abstract

Formation and dynamic control of strong coupling among cavities are essential to realize advanced functional photonic and quantum circuits. Especially for cavities at distant distance or arbitrary locations. Conventional approaches suffer from short coupling distance, poor controllability, fixed locations and low wavelength uniformity, significantly restricting the scalability of photonic and quantum networks. Here, we exploit the intrinsic advantages of optical bound state in the continuum (BIC) and demonstrate an all-in-one solution for long-range coupled cavities. BIC metasurface can support a series of finite-sized quasi-BIC microlasers at arbitrary locations. The quasi-BICs microlasers have the same wavelength and are inherently connected through BIC metasurface. Consequently, the coupling distances in experiment increase significantly from subwavelength to tens of micrometers. Such long-range interaction in BIC metasurface enables scaling to two-dimensional architectures and ultrafast control of internal laser actions, e.g., non-Hermitian zero-mode lasing. This research shall facilitate the advancement of scalable and reconfigurable photonic networks.

## Abstract (web summary)

This paper utilizes an optical BIC metasurface to achieve controllable long-range optical coupling, breaking through the limitations of traditional methods in coupling distance and reconfigurable tuning.

## Graphical abstract

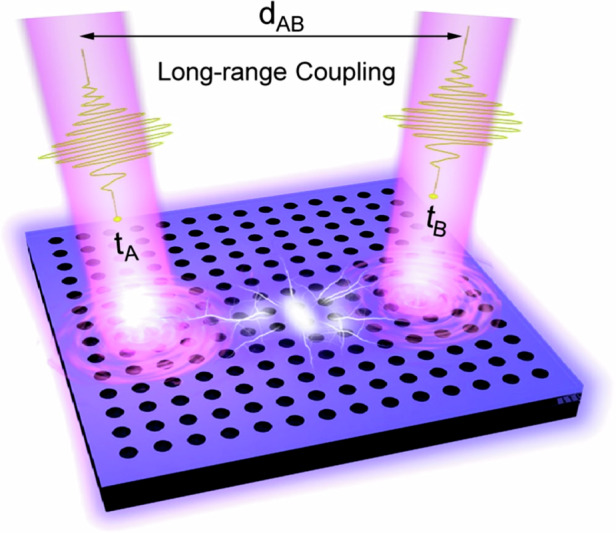

## Introduction

Coupled arrays of advanced photon sources are critical to advances in optical computing and quantum information processing^1–7^. Such arrays usually consist of a large number of micro- or nano-sized cavities linked with their mutual coupling. Over the past decades, there has been rapid progress in individual photonic devices. High quality (Q) factors, small effective mode volume, and the corresponding light-mater interactions have been intensively explored in both classical and quantum regimes^8–10^. Nonetheless, the scaling from discrete sites to large-scale photonic or quantum networks, is strongly hampered by the short interaction distance. Tight confinement of light in each resonator improves the performance of individual nanophotonic devices, but also strongly limits its coupling to adjacent nodes within a fraction of wavelength^5–7^. Several recent techniques utilizing zero-refractive index materials^11^, hyperbolic metamaterial^12^, and Weyl point in engineered nanostructures^13^ have demonstrated the theoretical potential to improve the coupling distance and its dynamical control. Practically, they still face the severe challenge of the trade-off between coupling range and strength^14^. One exception is the waveguide that can extend the interaction range far beyond evanescent field without compromising the strength^15,16^. However, conventional waveguides are limited to one-dimensional configuration, and the resonators involved typically have fixed locations and suffer from inevitable wavelength detuning, severely restricting the realization of reconfigurable and scalable networks.

In searching for strategies for controllable long-range coupling mechanism, we turn to the optical bound state in the continuum (BIC)^17^. BIC refers to the state that remains localized despite residing in the continuum spectrum of radiation. Optical BICs have been intensively studied in a variety of nanophotonic structures due to their extremely high quality (Q) factors and unprecedented capability in controlling the topological structures in momentum space^18–21^. In principle, BIC require the engineered nanostructures to be infinitely large in at least one dimension^17,22^. This requirement is generally regarded as a major drawback of BIC, seriously affecting the integration density and internal light-matter interaction of BIC devices. The inherent advantages of optical BIC in long-range interactions and coupled photonic arrays, however, have been simply overlooked for a very long time. Here, we take quasi-BIC microlaser as an example to demonstrate a new platform for controllable long-range coupling and scalable photonic circuits. We show that the BIC metasurface enables two-dimensional coupling with arbitrary resonator placement and uniform wavelengths, overcoming the constraints of coupling distance and strength in traditional methods.

## Results

### The working principle of distant coupling

**Fig. 1:**
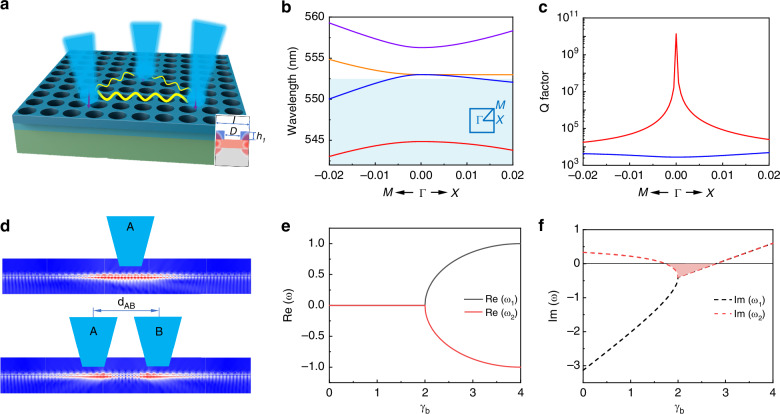
Interactions between quasi-BIC microlasers. **a** Schematic of the BIC metasurface. Inset shows the near-field mode profile in x-z plane. **b** Band structure of the BIC nanostructure. The gain spectrum is marked with a shadowed area. **c** Q-factors of resonances within the gain spectrum. **d** Schematic of long-range photon propagation (top) and long-range interaction (bottom) via BIC waveguide. (**e**, **f**) Show the real and imaginary parts of the eigenvalues of two coupled quasi-BIC resonators. For simplicity, we define ${\omega }_{0}=0$, ${\gamma }_{a}=4$, ${J}_{{ba}}={J}_{ab}=1$, ${\kappa }_{a}={\kappa }_{b}=3.4$. The lasing threshold is defined as *Im*(*ω*) = 0

The schematic of our metasurface is depicted in Fig. 1a. It is composed of an active membrane with a thickness *h*_*1*_ = 100 nm on a glass substrate. The membrane is covered with a 150 nm polymer film that is periodically patterned with square-lattice air holes. The lattice size and diameter of hole are *l* = 333 nm and *D* = 167 nm, respectively. For the case of infinite period, numerical simulation (see Methods and Supplementary Note-1) reveals that two resonances with transverse magnetic (TM, where E is perpendicular to the plane) polarization appear within the gain spectrum of active layer (shadowed area in Fig. 1b). The lower branch corresponds to the well-known symmetry-protected BIC at Γ-point. The coherent destruction of scattering waves confines electromagnetic field as guiding waves in the active membrane. As a result, the Q factor increases exponentially approaching Γ-point and reaches a maximal value of 10^10^ (Fig. 1c).

The ideal BIC degrades to quasi-BIC when the metasurface is excited by a laser beam of finite size (Supplementary Note-1)^19^. The electromagnetic waves at the BIC wavelength are amplified and lasing in the pump regions. Such quasi-BIC microlasers produce vector beams in the vertical direction. Their in-plane radiations are less confined and propagate to distant places through the non-radiative BIC mode (top panel in Fig. 1d). The situation becomes very intriguing when two or more pump beams are applied (bottom panel in Fig. 1d). Different from conventional photonic systems, the quasi-BIC microlasers are defined by the external excitations and can be generated at arbitrary locations of BIC metasurface. On one hand, the laser systems can be easily reconfigured. On the other and, their operation wavelengths are determined by the lattice size and are naturally the same. Such quasi-BIC microlasers are inherently linked with the non-radiative BIC mode. Then the trade-off between coupling range and strength is simply broken and a complex network can be constructed in the two-dimensional BIC metasurface. Therefore, BIC metasurface can be an ideal platform scalable and reconfigurable photonic circuits.

For simplicity, we consider the interaction between two quasi-BIC microlasers. When the excitation is not far above the laser threshold, the gain saturation is absent and the interaction can be expressed as:

1a $$i\frac{da}{{dt}}={\omega }_{0}a+{i(\gamma }_{a}-{\kappa }_{a})a+{J}_{{ba}}b$$

1b $$i\frac{db}{{dt}}={\omega }_{0}b+{i(\gamma }_{b}-{\kappa }_{b})b+{J}_{{ab}}a$$

Here $a$ and $b$ are the amplitudes of two quasi-BICs with the same angular frequency of ${\omega }_{0}$. ${\gamma }_{a,b}$ and ${\kappa }_{a,b}$ represent the gain and loss coefficients, respectively. The coupling is mediated by the BIC waveguide and its coefficients are defined as ${J}_{{ab}}$ and ${J}_{{ba}}$ (here we set ${J}_{{ab}}={J}_{{ba}}=J$). Then the eigenvalues are given as:

2 $${\omega }_{\!\pm }={\omega }_{0}+i({\gamma }_{{avg}}-k)\pm \sqrt{{J}^{2}-{(\Delta \gamma )}^{2}}$$

where $\Delta \gamma =\left({\gamma }_{a}-{\gamma }_{b}\right)/2$, ${\gamma }_{{av}g}=\left({\gamma }_{a}+{\gamma }_{b}\right)/2$, and ${k}_{a}={k}_{b}=k$. The entire system operates as a single quasi-BIC microlaser when ${\gamma }_{a}$ is tuned only and ${\gamma }_{b}$ is fixed as zero. The coupling occurs when the gain coefficients ${\gamma }_{a}$ is kept at above the threshold and ${\gamma }_{b}$ becomes nonzero. With the increase of ${\gamma }_{b}$, the imaginary parts of eigenvalues (*Im*(*ω*_*±*_)) approach one another and merge at $\Delta \gamma =J$, whereas the real parts (*Re*(*ω*_*±*_)) remain at their initial values and bifurcate (Fig. 1e, f). Then the coupled quasi-BIC microlasers can be considered as a quasi-parity-time symmetric system and reaches the exceptional point (EP) at $\Delta \gamma =J$^23–29^.

In the BIC metasurface, only the waves propagating to beam B are involved in the mode interaction (Supplementary Note-1). The other outgoing waves only contribute to optical loss and give a relatively large loss factor *k*. As a consequence, EP can appear at relatively large *γ*_*avg*_ and below the threshold line. One example is illustrated in Fig. 1f). The imaginary part of the lasing mode (*Im*(*ω*) > 0) decreases with the increase of gain coefficient at beam B (${\gamma }_{b}$) and eventually both *Im*(*ω*_*±*_) are below the threshold line (the shadowed region). This process corresponds to the well-known phenomena of lasing self-termination and even lasing death^30–33^. Further increases of ${\gamma }_{b}$ can make the coupled system lase again (*Im*(*ω*_*±*_) > 0). Under such a situation, the bifurcation in real parts (*Re*(*ω*_*±*_)) lead to an obvious mode splitting in emission spectrum (Fig. 1e). Therefore, the lasing self-termination and mode splitting can be two important criteria for exploring and determining the interaction of quasi-BIC microlasers.

### Experimental demonstration of long-range coupling at BIC

**Fig. 2:**
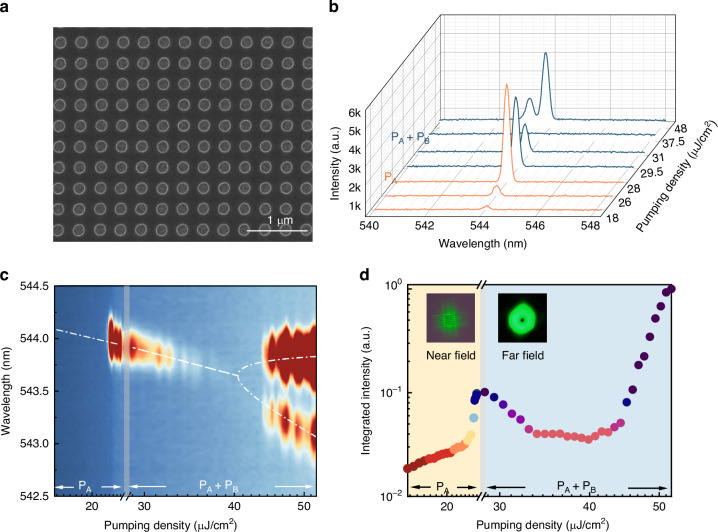
Long-range coupling between dual quasi-BIC microlasers. **a** Top-view SEM image of the perovskite-based BIC metasurface, featuring a square-lattice nanohole array patterned in ZEP520A resist. **b** Emission characteristics under varying pumping intensity: the yellow solid lines display the emission spectrum of an isolated quasi-BIC microlaser, while the blue solid lines show spectral evolution with increasing pumping intensity of beam-B (with beam-A fixed at 1.2 P_th_). **c** Two-dimensional spectral mapping comparing single-beam and dual-beam pumping conditions. The theoretical fit employs these parameters: *Re*(*ω*_*0*_) = 3462 THz, ${\gamma }_{a}$ = 16 THz, 0 < ${\gamma }_{b}$ < 16 THz, *J* = 4 THz, *k* = 14 THz. **d** Integrated output intensity versus pumping intensity for both single-beam (yellow) and dual-beam (blue) operation modes. Insets present corresponding near-field and far-field profiles of the quasi-BIC microlaser

Based on the above analysis, we have fabricated the designed BIC metasurfaces on a K9 glass substrate with a standard electron-beam (E-beam) lithography process (see Methods and Supplementary Note-2)^34,35^. Periodic air holes are patterned in a 150 nm E-beam resist E-beam resist (ZEP520A) and the optical gain is provided by the underneath Quasi-2D perovskite (N_2_F_8_) film with a thickness of 100 nm. The overall sample size is 100 × 100 μm^2^. Figure 2a depicts the top-view scanning electron microscope (SEM) image of the BIC metasurface. Similar to the thicknesses, both the hole diameter and the lattice size follow the design well. Then BIC mode and the corresponding lasing actions can be expected.

The optical characteristics of BIC metasurface are explored by exciting it with a frequency doubled femtosecond laser under a home-made pump-probe system (see Methods and Supplementary Note-3). The yellow solid lines in Fig. 2b summarize the recorded emission spectra under different pump fluences of beam A only (*P*_*A*_, here *P*_*B*_ = 0). The diameter of excitation beam is fixed at *D* = 5 μm in this research. Broad photoluminescence peak is achieved at low pump fluence and a sharp peak emerges when *P*_*A*_ is around 25 μJ cm^-2^. The sharp spike slightly shifts to shorter wavelength due to the band filling effect (left panel in Fig. 2c). Its intensity increases dramatically and dominates the spectrum at higher pump fluence. Such behaviors are associated with the slope change in log-log plot of light-light curve (left panel in Fig. 2d) and a donut beam appears in far field (inset in Fig. 2d). All these observations confirm the quasi-BIC microlaser with a threshold of *P*_*th*_ = 25 μJ cm^-2^ in our metasurface. Then the location of excitation beam varies and the corresponding lasing actions are recorded. The good uniformity in both lasing wavelength and threshold are confirmed experimentally (Supplementary Note-3). The quasi-BIC laser mostly propagates in-plane along *x*- and *y*- directions (inset of Fig. 2d) and has restricted loss. Therefore, BIC metasurface can be an ideal platform to explore the proposed distant interaction.

Then we fix the power of beam A at *P*_*A*_ = 1.2 *P*_*th*_ and turn on beam B, which is 30 μm (center to center distance, *d*_*AB*_) away from beam A along *x*-direction. While two beams are widely separated (~50 λ), we can still see the changes in lasing characteristics with the increase of pump power *P*_*B*_. The emission wavelength shifts to shorter wavelength and the emission intensity decreases with the total pump fluence (*P*_*total*_ = *P*_*A*_ + *P*_*B*_) (blue solid lines in Fig. 2b). When *P*_*B*_ is above 0.6 *P*_*th*_, the lasing mode disappears and only a broad photoluminescence remains. The broad photoluminescence peak above the laser threshold is well preserved over a large power range of *P*_*B*_ from 0.6 *P*_*th*_ to 0.85 *P*_*th*_ (1.8 *P*_*th*_ < *P*_*total*_ < 2.05 *P*_*th*_). With a further increase of *P*_*B*_, the lasing peak re-emerges and a doublet can be clearly seen in the emission spectrum. The wavelengths and intensity of lasing modes are summarized in right panels of Fig. 2c, d. The reduction in intensity, disappearance of lasing mode, and mode spitting can be more clearly seen, consistent with the theoretical model of lasing self-termination very well. Due to the relatively large power difference between the two beams, we note the exceptional point (EP) was submerged within the lasing self-termination region. In fact, the virtual EP in the PT-symmetric system can be approached by adjusting the relative power difference between the two beams (Supplementary Note-5). Meanwhile, the separation distance *d*_*AB*_ = 30 μm is orders of magnitude larger than the carrier diffusion length in perovskite film^36^. The long-range interaction between two quasi-BIC microlasers can thus be confirmed. Besides, we found that even if both pump beams are below the threshold, coupled lasing emission can still occur when their distances are close (Supplementary Note-4).

**Fig. 3:**
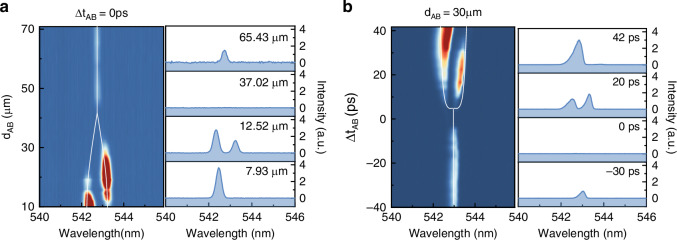
Tunable long-range coupling between two quasi-BICs. **a** Distance-dependent coupling control. *Left:* Two-dimensional spectral map showing emission as a function of beam separation (*d*_*AB*_). *Right:* Emission spectra at selected distances *d*_*AB*_ = 7.93 μm, 12.52 μm, 37.02 μm, 65.43 μm. **b** Delay-dependent coupling dynamics. *Left:* Two-dimensional spectral map versus time delay (Δ*t*_*AB*_) between excitation beams. Dashed curves represent theoretical fits based on coupled-mode theory. *Right:* Emission spectra at key delays (Δ*t*_*AB*_ = -30 ps, 0 ps, 20 ps, 42 ps)

Quasi-BIC microlasers are defined by excitation beam at arbitrary locations of metasurface. Then the coupled system can be reconfigured by changing the separation distance between two beams and the maximal coupling distance can be experimentally determined. Here two beams reach the sample simultaneously and their pump fluences are 1.2 *P*_*th*_ and 0.8 *P*_*th*_, respectively. We experimentally vary the position of beam B along x-direction and all the results are summarized in Fig. 3a. Two pump beams are obviously separated when *d*_*AB*_ is larger than 9 μm. Under such a situation, strong coupling occurs and a lasing doublet can be observed. With the increase of *d*_*AB*_, two lasing modes approach one another and gradually disappear at *d*_*AB*_ ≥ 30 μm. The lasing peak reappears at *d*_*AB*_ > 45 μm. It becomes a single mode again and its intensity gradually increases with *d*_*AB*_. All these observations are also attributed to the mode interaction. In experiment, the propagation in perovskite film is affected by the material loss and exponentially decay with the distance. Taking the position dependent *J* into accounted, the eigenvalues have been calculated and plotted as dashed lines in Fig. 3a. Both the doublet at short distance and the lasing self-termination phenomenon can be reproduced (Supplementary Note-4). When the separation distance *d*_*AB*_ is above 60 μm, we find that the emission intensity of coupled system saturates at the value of a single microlaser with a pump fluence of 1.2 *P*_*th*_. This shows that the lasing self-termination effect is negligibly small at *d*_*AB*_ > 60 μm. Then we know that the quasi-BIC microlasers can at least interact each other with a separation distance up to ~ 110 λ. Notably, since the finite-size effect influences the quality factor of quasi-BIC, also the coupling distance. We observed that a larger spot size leads to a longer coupling distance between the quasi-BIC resonators (Supplementary Note-1 and Note-4).

Another important feature between long-range interaction from evanescent coupling is its dynamic controllability. According to Eq. (2), the eigenvalues of hybrid states can also be controlled with the difference in gain coefficients of two quasi-BICs ($\Delta \gamma =\left({\gamma }_{a}-{\gamma }_{b}\right)/2$). In principle, the gain coefficients ${\gamma }_{a}$ and ${\gamma }_{b}$ are closely related to the population inversion. For the case of ultrafast excitation, it is essential to note that ${\gamma }_{a}$ and ${\gamma }_{b}$ are no longer time invariant. Instead, they change as a function of time due to the evolution of population inversion after the ultrafast excitation (Supplementary Note-5). As a consequence, even though the fluences of two excitation beams are fixed, the difference in gain coefficient ($\Delta \gamma$) can still be time dependent if they reach the sample at different time. This simply triggers the possibility of dynamically controlling the distant coupling with a delay time between two beams (Δ*t*_*AB*_ = *t*_*B*_ – *t*_*A*_, the negative value means that beam B arrives earlier).

Then we verify this possibility in experiment. Here two beams are separated with a distance of *d*_*AB*_ = 30 μm and their fluences are fixed at 1.2 *P*_*th*_ and 0.8 *P*_*th*_, respectively. The delay time Δ*t*_*AB*_ is controlled by a delay line (Supplementary Note-3). The emission spectra at different Δ*t*_*AB*_ have been recorded and analyzed in Fig. 3b. The gain coefficient ($\Delta \gamma$) increases as a function of Δ*t*_*AB*_. According to Eq. (2), the interaction between two modes increases too and the lasing self-termination phenomenon occurs. This analysis has been experimentally confirmed with the trend of laser intensity. It decreases with the increase of Δ*t*_*AB*_ at the beginning. Further increase of Δ*t*_*AB*_ can fully suppress the lasing mode at Δ*t*_*AB*_ = −4.5 ps, leading to the well-known lasing self-termination effect. Similarly, the lasing mode reappears at Δ*t*_*AB*_ ≥ 5 ps and an obvious laser doublet can be seen. Therefore, we confirm that the long-range interaction in our metasurface can be dynamically and ultrafast controlled. The corresponding emission spectrum can be switched from single-mode laser emission to photoluminescence and mode splitting within a delay time around 10 ps.

### Multiple coupled quasi-BIC microlasers and the ultrafast control

**Fig. 4:**
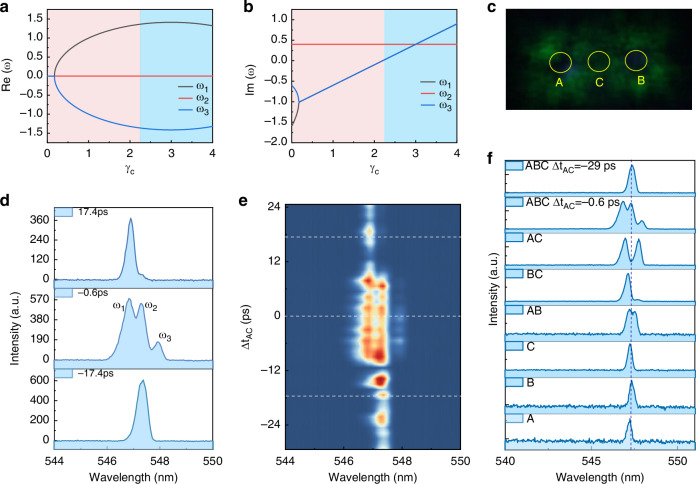
Non-Hermitian Zero mode formed by three coupled quasi-BICs. **a**, **b** Show the real and imaginary part of the eigenvalues of three-body coupling system, respectively. The calculation parameters are set as: *J*_*ab*_ = 0, *J*_*ac*_ = *J*_*ca*_ = *J*_*cb*_ = *J*_*bc*_ = 1, *k*_*a*_ = *k*_*b*_ = 3.4, *k*_*c*_ = 2.6, ${\gamma }_{a}={\gamma }_{b}$ = 3.8 The zero-mode lasing region is indicated by the pink shaded area. **c** Near field image of the zero-mode lasing pumped by three collinear beams after filter the pumping beam, the pump intensity here are set as *P*_*A*_ = *P*_*B*_ = *P*_*C*_ = 1.1 *P*_*th*_, the delay times Δ*t*_*AB*_ = 0 ps, *d*_*AC*_ = *d*_*BC*_ = 15 μm. **d** Typical emission spectrum at Δ*t*_*AC*_ = −17.4 ps, −0.6 ps, and 17.4 ps. **e** The whole evolution of emission spectrum with the increase of Δ*t*_*AC*_ when three beams arranged as indicated in (**c**). **f** Emission spectrum of the sample with different combinations of three pumping beams

One BIC metasurface is able to support a series of quasi-BIC microlasers with near-zero detuning at arbitrary locations. This characteristic associated with the long-range reconfigurable interaction, enables the coupling between multiple nodes. In this sense, although there is no topological origin in the structure, the long-range coupling properties of BIC metasurface allow us to conveniently study more non-Hermitian (NH) topological optical phenomena. Compared to topologically protected edge states, the NH zero modes protected by non-Hermitian particle-hole symmetry are not restricted to interfaces and can be excited in different regions of a coupled photonic array, including both the bulk and edges^37^. In other words, by pumping different sites of the BIC metasurface, we can generate the zero modes in coupled quasi-BICs with designed coupling coefficients. Due to experimental setup limitations, here we take the three-site interaction as examples to illustrate this potential. In principle, the system can be described with a 3 × 3 non-Hermitian Hamiltonian:

$$H=\left(\begin{array}{ccc}{\omega }_{0}+i({\gamma }_{a}-{k}_{a}) & {J}_{{ab}} & {J}_{{ac}}\\ {J}_{{ba}} & {\omega }_{0}+i({\gamma }_{b}-{k}_{b}) & {J}_{{bc}}\\ {J}_{{ca}} & {J}_{{cb}} & {\omega }_{0}+i({\gamma }_{c}-{k}_{c})\end{array}\right)$$

where ${\gamma }_{i}$ and ${k}_{i}\,(i=a,b,c)$ are the gain coefficients and loss factors of each resonator, ${J}_{ij}(i,j=a,b,{c;i}\ne j)$ are the coupling factor between different resonators. We first consider a simple co-linear configuration of three-body interaction, where beam C locate in the middle between A and B. Figure 4a, b show the eigenvalues as a function of ${\gamma }_{c}$ (See details in Supplementary Note-6). When ${\gamma }_{c}$ < 2.2 (${\gamma }_{c}$ < ${k}_{c}$, below the threshold), only imaginary part of ω_2_ exceeds zero, and the real part of *ω*_2_ situated between *ω*_1_ and *ω*_3_, indicating that the system entered in the zero-mode lasing region. With the increase of ${\gamma }_{c}$, the real parts of the eigenvalues separate into three distinct values at large ${\gamma }_{c}$, while the imaginary parts of all eigenvalues remain positive, indicating the occurrence of three-mode lasing.

In contrast to evanescent coupling between traditional optical cavities, the long interaction distances allow individual control of pump intensity and their relative delay time (Δ*t*_*AB*_, Δ*t*_*AC*_, Δ*t*_*BC*_), enabling the precise control of zero-mode lasing action. In experiment, we set the pump intensity of the three beams as *P*_*A*_ = *P*_*B*_ = *P*_*C*_ = 1.1 *P*_*th*_, and their separation distances are set as *d*_*AC*_ = *d*_*BC*_ = 15 μm. The coupled system produces three lasing peaks when three beams pump the sample simultaneously, i.e., −10 ps < Δ*t*_*AC*_ < 10 ps, as seen in Fig. 4d, e. Then we adjust the time delay between the middle beam (beam C) and the other two beams (Δ*t*_*AB*_ = 0 ps and Δ*t*_*AC*_ = Δ*t*_*BC*_) to explore the lasing behaviors. The whole emission spectrum is summarized in Fig. 4e. For the case of Δ*t*_*AC*_ < −10 ps, the gain of beam C (${\gamma }_{c}$) is small since beam C pumping the sample earlier than the other two beams, consequently, the system entered in the zero-mode lasing region. As shown in bottom panel of Fig. 4d, e, three lasing peaks collapsed into the central single mode. When beam C pumping the sample later, (i.e., Δ*t*_*AC*_ > 10 ps), the excited-state carriers generated by beam C gradually decay, the dominant interaction occurs between A and B. It can be seen that only the split double peaks appear in the spectrum, and the spectrum exhibits a blue shift due to the band renormalization caused by the gain carriers generated by beam C.

In addition, to rule out the emission spectrum is caused by large detuning between the resonators, we further verified the emission spectra of the three beams when pumped individually and coupled in pairs, as shown in Fig. 4f. When the three beams are pumped individually (labeled as A, B, C in the figure, respectively), only single-mode lasing emission exhibited, while when two beams pumped, the system exhibits a split double peak, indicating the strong coupling between any two resonators. Notably, the splitting is relatively small for beam A and beam B pumped simultaneously (labeled as AB in the figure) compared to the situation of AC and BC due to the furthest distance between A and B. Furthermore, when the three beams are pumped simultaneously (labeled as ABC, Δ*t*_*AC*_ = −0.6 ps), the system shows two additional peaks on either side of the original single-mode peak, forming three-mode lasing. When C is pumped earlier than A and B (labeled as ABC, Δ*t*_*AC*_ = −29 ps), the coupled three-mode laser degenerates into a single-mode laser, and the position of the single-mode lasing peak almost coincides with the original single-beam pumping, once again confirmed that the system lasing at zero-mode.

Finally, since the symmetry protected BIC resonant along both Γ-M and Γ-X direction near Γ point, the two-dimensional BIC metasurface allows for more complex coupling compared to one-dimensional waveguides. We show that when the three sites are not collinear, the system can still maintain a coupled state and achieve dynamic switching between three-body coupling and two-body coupling by adjusting the delay (Supplementary Note-7). All these experimental results demonstrate the advantage of distant coupling in a BIC metasurface, which facilitates the realization of large coupled laser arrays. Notably, for more complex networks, additional direction coupling may be required. In practice, our etching-less fabrication process can be extended to higher-order symmetric structures, such as C_6V_ symmetric structures (Supplementary Note-1). This advancement holds great potential for large-scale optical computing and reconfigurable photonic neural networks.

## Discussion

In summary, we demonstrate that BIC metasurface can function as an ideal platform for the controllable long-range interaction. The uniform lasing wavelength and two-dimensional BIC metasurface greatly increase the interaction distance up to tens of micrometers, which introduces the dynamic control of mode coupling and enables the construction of scalable and reconfigurable photonic circuits. Our concept can be extended to a large array of coupled microlasers and even passive cavities. It is thus able to empower the researches of topological photonics^38,39^, supersymmetry^40,41^, reservoir computing^2,3^, and quantum network^42^ with precisely controllable coupling constants.

Due to experimental setup limitations, in this paper we only demonstrated the three-site interaction as an example to illustrate long-range interactions among multiple nodes. Future work should overcome limitations in material losses and adopt scalable fabrication techniques like nanoimprinting to achieve longer coupling distances while maintaining strong interactions, thereby enabling scaling to larger networks. Additionally, integration with topological photonic structures may create much more functional hybrid systems combining our platform’s reconfigurability with topological protection. Ultimately, this research represents a new paradigm for scalable coupled systems, bridges the gap between nanophotonic integration and large-scale programmable networks. and shall revolutionize the optical computing and quantum information processing.

## Materials and methods

### Numerical simulation

The band structures, Q-factors, and the corresponding field pattern were calculated with a finite-element method using commercial software (COMSOL Multiphysics). Periodic boundary conditions are applied in the *x*- and *y*- directions to mimic the infinite large periods. Perfectly matched layers are used the *z* direction to fully absorb the outgoing waves. Optical constants of the active layer and top polymer are obtained from experimental results of Quasi-2D perovskite and ZEP520A resist, which are measured by ellipsometry. The refractive index of glass substrate is set as 1.45.

### Sample Fabrication

The BIC metasurface is fabricated on a 13 nm ITO coated glass substrate by a combined process of spin-coating of Quasi-2D perovskite film and electron beam lithography. The precursor solutions of N_2_F_8_ ((NMA)_2_FA_n-1_Pb_n_Br_3n+1_, *n* = 8) was prepared by solving a 25% molar ratio of 1-naphthylmethylamine bromide (NMABr) into a mixture of HC(NH_2_)_2_Br (FABr) and PbBr_2_ (1:1 ratio) in DMF at 0.4 M and stirred at 60 °C for 12 h. The mixtures were then spin-coated on the ITO layer at 5,000 r.p.m. for 30 s. During spin coating, 0.3 ml of ethyl acetate was dropped onto the perovskite precursor layer. The substrates were baked on a hotplate at 85 °C for 15 min. Then 100 nm ZEP520A was spin-coated onto the film and patterned with electron-beam lithography (Raith e-LINE). After developing in N50 for 60 s, the metasurface was eventually achieved.

### Optical characterization

The sample was optically excited by a frequency-doubled Ti:Sapphire laser (400 nm, repetition rate 1 kHz, pulse width 100 fs). The incident laser was first divided into three beams, two of which passed through two delay lines respectively. Three beams were then combined and focused onto the surface of the sample using an objective lens (40X, NA = 0.65). The spatial positions and delay times of three beams are controlled by spatial deviations and delay lines, respectively. The experimental details have been summarized in Supplementary Note-3.

## Supplementary information

Supplementary Information for Dynamically Tunable Long-range Coupling Enabled by Bound State in the Continuum

## Data Availability

The data that support the findings in this study is available from the corresponding authors upon reasonable request.
