# Author Correction: Dynamically Tunable Long-range Coupling Enabled by Bound State in the Continuum

**DOI:** 10.1038/s41377-026-02261-1

**Published:** 2026-04-03

**Authors:** Haijun Tang, Can Huang, Yuhan Wang, Xiong Jiang, Ruiheng Jin, Yue Cui, Shumin Xiao, Qinghai Song

**Affiliations:** 1 Ministry of Industry and Information Technology Key Lab of Micro-Nano Optoelectronic Information System, Guangdong Provincial Key Laboratory of Semiconductor Optoelectronic Materials and Intelligent Photonic Systems, Harbin Institute of Technology, Shenzhen, China; 2 Pengcheng Laboratory, Shenzhen, China; 3 National Key Laboratory of Science and Technology on Advanced Composites in Special Environments, Harbin Institute of Technology, Harbin, China; 4 Quantum Science Center of Guangdong-Hongkong Macao Greater Bay Area, Shenzhen, China; 5 Heilongjiang Provincial Key Laboratory of Advanced Quantum Functional Materials and Sensor devices, Harbin Institute of Technology, Harbin, China; 6 Collaborative Innovation Center of Extreme Optics, Shanxi University, Taiyuan, Shanxi China

**Keywords:** Nanophotonics and plasmonics, Lasers, LEDs and light sources

Correction to: *Light: Science & Applications*

10.1038/s41377-025-01975-y, published online 18 August 2025

In the version of this article originally published, there were unit errors in the pumping power density reported in the main text and the supplementary information. Specifically, the following changes were made:

In the main text: The “Experimental demonstration of long-range coupling at BIC” section on page 3: “250 nJ cm^−2^” modified as “25 μJ cm^−2^.”

The corresponding unit in the Figure 2 has also been modified, the correct figure 2 is shown below:

Incorrect figure 2
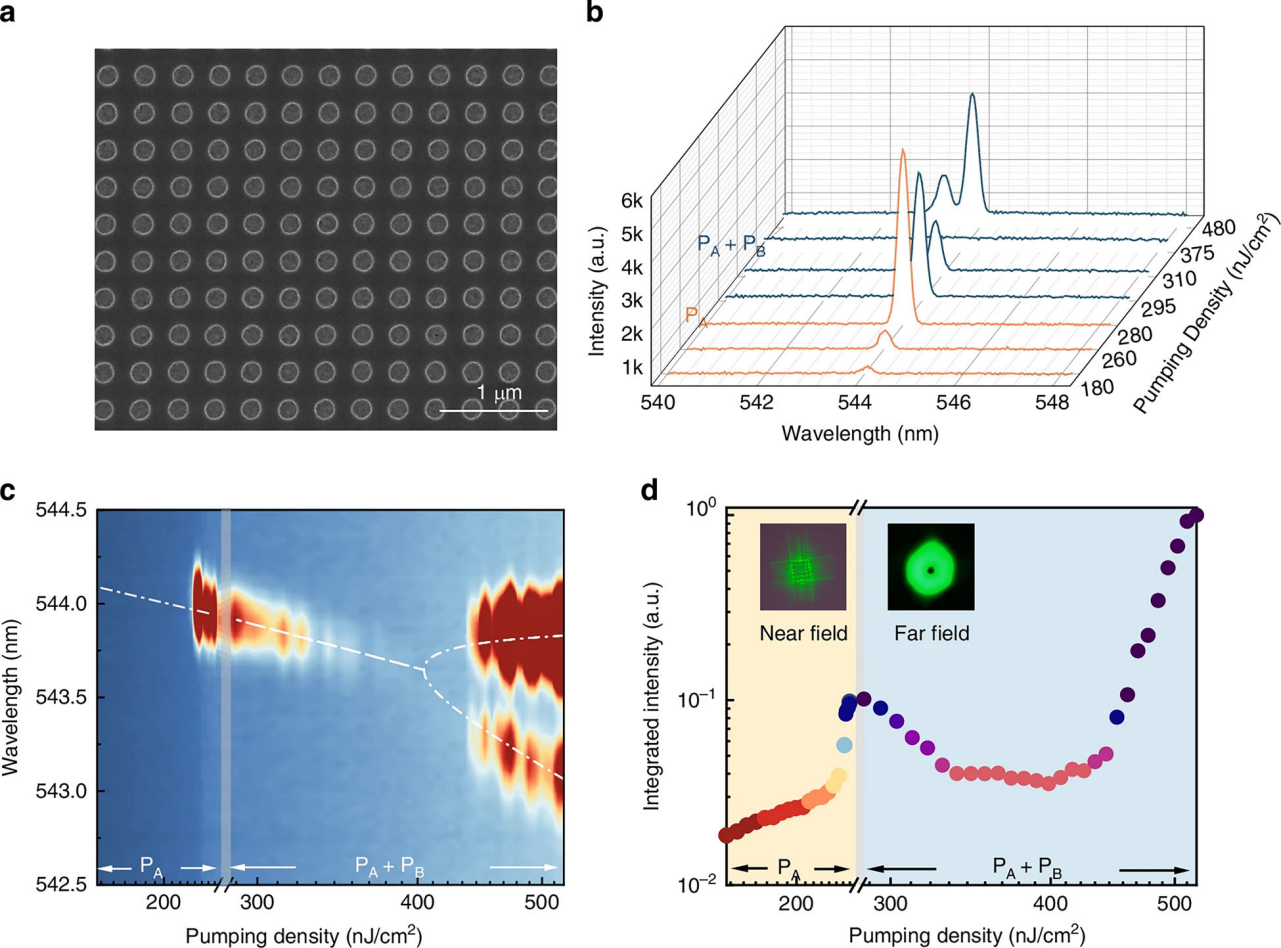


Correct figure 2
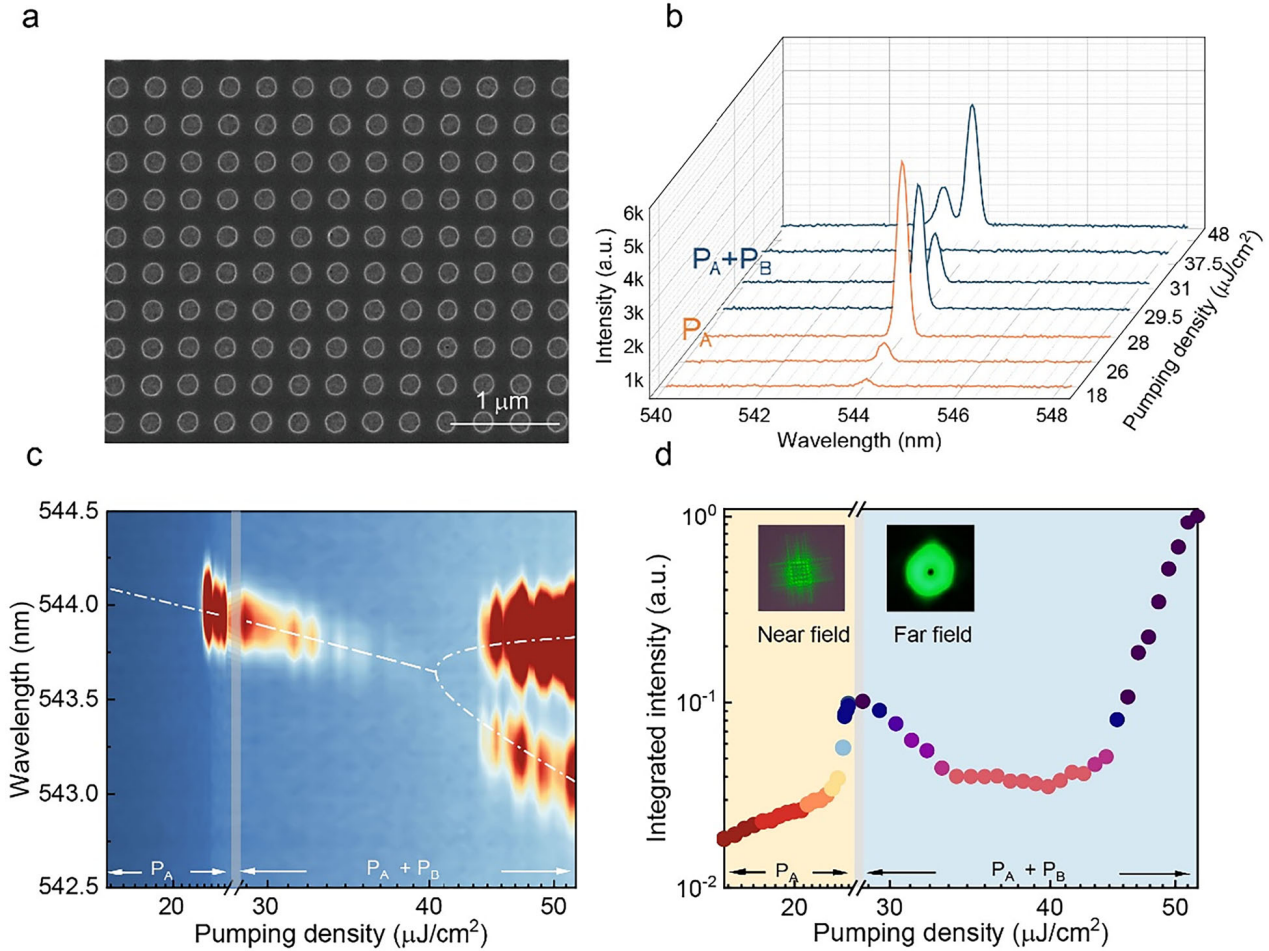


The original paper has been updated.

## Supplementary Information

Supplementary Information

