# Supplementary material for: Dynamically tunable long-range coupling enabled by bound state in the continuum
Source: Light Sci Appl. 2025 Aug 18;14:278. doi: 10.1038/s41377-025-01975-y (PMC12361451; doi:10.1038/s41377-025-01975-y)
Supplement: Supplementary file 1 — Supplementary Information for Dynamically Tunable Long-range Coupling Enabled by Bound State in the Continuum [file 41377_2025_1975_MOESM1_ESM.docx]

**Supplementary Information for**

**Dynamically Tunable Long-range Coupling Enabled by Bound State in the Continuum**

Haijun Tang^1, 2, 3 †^, Can Huang^1, 4, 6, †, #^, Yuhan Wang^1^, Xiong Jiang^1^, Ruiheng Jin^1^, Yue Cui^1^, Shumin Xiao^1, 2, 3, 4, 5, 6^, Qinghai Song^1, 2, 4, 5, 6 *^

^1^ Ministry of Industry and Information Technology Key Lab of Micro-Nano Optoelectronic Information System, Guangdong Provincial Key Laboratory of Semiconductor Optoelectronic Materials and Intelligent Photonic Systems, Harbin Institute of Technology, Shenzhen 518055, China.

^2^ Pengcheng Laboratory, Shenzhen 518055, China.

^3^ National Key Laboratory of Science and Technology on Advanced Composites in Special Environments, Harbin Institute of Technology, Harbin 150080, China.

^4^ Quantum Science Center of Guangdong-Hongkong Macao Greater Bay Area, Shenzhen 518055, China.

^5^ Collaborative Innovation Center of Extreme Optics, Shanxi University, Taiyuan 030006, Shanxi, China.

^6^ Heilongjiang Provincial Key Laboratory of Advanced Quantum Functional Materials and Sensor devices, Harbin Institute of Technology, Harbin 150001, China

^†^ These authors contribute equally to this work.

Corresponding authors:

* qinghai.song@hit.edu.cn; # huangcan@hit.edu.cn;

**Context**

[**Note-1. Numerical simulation 3**](#_Toc188786345)

[**Note-2. Sample preparation 9**](#_Toc188786346)

[**Note-3. Optical setup for lasing experiments 11**](#_Toc188786347)

[**Note-4. Long range interaction at BIC 15**](#_Toc188786348)

[**Note-5. Dynamic control of distant coupling 18**](#_Toc188786349)

[**Note-6. Zero mode in the three coupled quasi-BICs 21**](#_Toc188786350)

[**Note-7. Distant interactions between multiple quasi-BICs 25**](#_Toc188786351)

## Note-1. Numerical simulation

**Band structure of BIC metasurface**

The band structures and quality (Q) factors of the BIC metasurface are calculated with a finite-element method based commercial software (COMSOL Multiphysics). The periodic boundary condition is applied in x and y direction to mimic the infinitely large sample sizes. Perfectly matched layers are employed along the z direction to absorb the outgoing waves. The optical constants of lead halide perovskite were obtained from ellipsometry measurement (see Fig. S6 below). The refractive indices of glass substrate and polymer grating are fixed at n = 1.45 and n =1.54 for the designed lasing wavelength, respectively. The environment is defined as air with n = 1. The eigen-frequency solver in COMSOL was used to calculate the eigenfrequency. The simulation produces complex valued eigenfrequency (*ω*). Then the resonant frequency and Q factor can be achieved from *Re*(*ω*) and Q *= Re*(*ω*)*/|*2*Im*(*ω*)*|*, respectively. With the gradual change on incident angle, the band structure and Q-factors can thus be obtained. All the numerical results are summarized in Fig. S1. It is easy to see that both transverse magnetic (TM) polarization and transverse electric (TE) polarization can form the BICs with extremely high Q factors. Interestingly, there is only one mode (green line in Fig. S1(a)) lies in the gain spectral range of lead halide perovskite (shadowed area in Fig. S1(a)). Therefore, we confirm that the non-etching design in the main text can produce a single mode lasing emission. All the other modes are restricted either by the low Q factors or low gain coefficient.


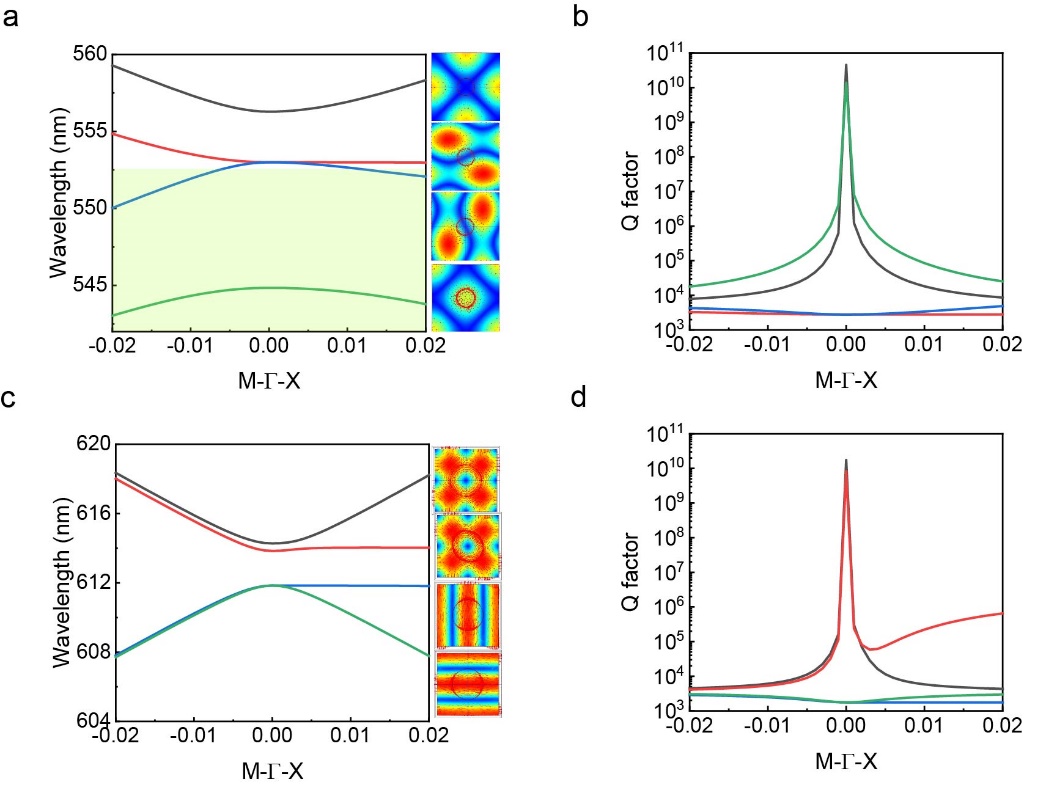


**Figure S1.** Band structure and corresponding Q-factors of the BIC structure. (**a**). Dispersion curve of TM mode in the perovskite-based BIC nanostructure, the insets show the corresponding near field distribution. (**b**) Corresponding Q-factors of the eigenstates in (a). (**c**). Dispersion curve of TE mode in the perovskite-based BIC nanostructure, the insets show the corresponding field distribution. (**d**) Corresponding Q-factors of the eigenstates in (c).

For more complex networks, additional direction coupling may be required. In practice, our etching-less fabrication process can be extended to higher-order symmetric structures, such as C_6V_ symmetric structures. As shown in Fig. S2. It can be seen that the high-Q BIC resonance mode can be formed in the etching-less structure. Therefore, coupling in more directions can actually be achieved by designing structures with higher-order symmetry.


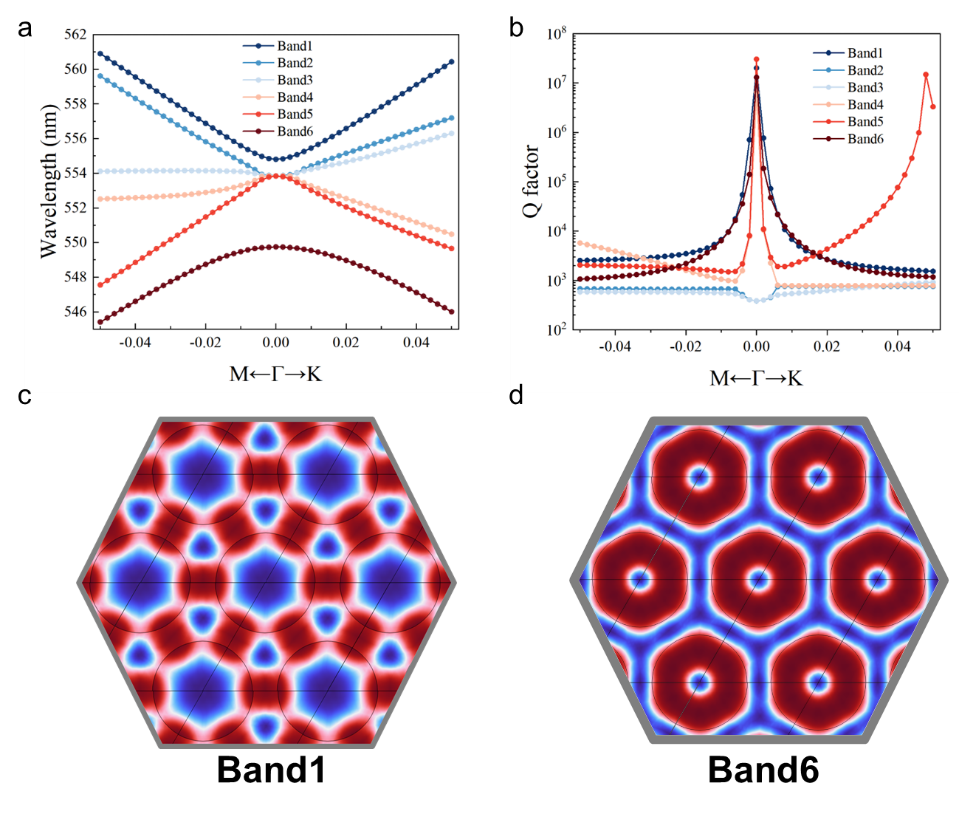


**Figure S2.** **Higher-order etching-less BIC structure.** (**a**) Band structure of the C_6v_ BIC nanostructure. (**b**) Q-factors of resonances corresponding to (a). (**c**) and (**d**) show the electrical field distribution of band 1 and band 6 in (a).

**Quasi-BIC lasing mode in BIC metasurface**

The simulation of BIC modes in Fig. S1 requires an infinite number of periods (sample size). It is well known that the BIC will degrade to quasi-BIC with quite limited Q factors when the size of metasurface is finite. The Q value drops exponentially and is only a few hundred when the device size approaches ten or a few micrometers. Such a requirement on infinite size is typically considered as a drawback of BIC since the real sample always has a boundary and large device size spoils the system integration seriously. In the main text, we reveal that the infinite sized BIC metasurface itself can be considered as a platform for photonic circuits, which are composed of numerous finite-sized quasi-BIC modes linked with BIC waveguide.

Here we will show the formation of quasi-BIC microlasers. The case of laser is different from a passive cavity. The external excitation and the gain materials are equally important. In other words, both the light confinement and the area of population inversion can greatly affect the eventual lasing characteristics. When the BIC metasurface is partially excited, the resonant modes within the pumped area experience the optical gain and reach the lasing threshold. The optical confinement of the pumped region is only realized with the conventional distributed feedback of the periodic structures and relatively low. For simplicity, we take the one-dimensional BIC as an example to illustrate this type of quasi-BIC microlasers. Similar to the main text, the BIC metasurface is realized by patterning one-dimensional grating on a lead halide perovskite film. All the refractive indices and the structural thicknesses are the same as the main text. The lattice size and the filling factor of the grating are 333 nm and 0.5, respectively. The entire BIC device has a finite size of 10 μm and two in-plane side-facets of the sample enter the perfect matching layers to absorb the outgoing waves and avoid the backward waves. Without optical pumping, the device gives a Q factor of 465. With the increase of optical gain, we can see the transition from a lossy cavity to optical gain (imaginary part of frequency is above zero).


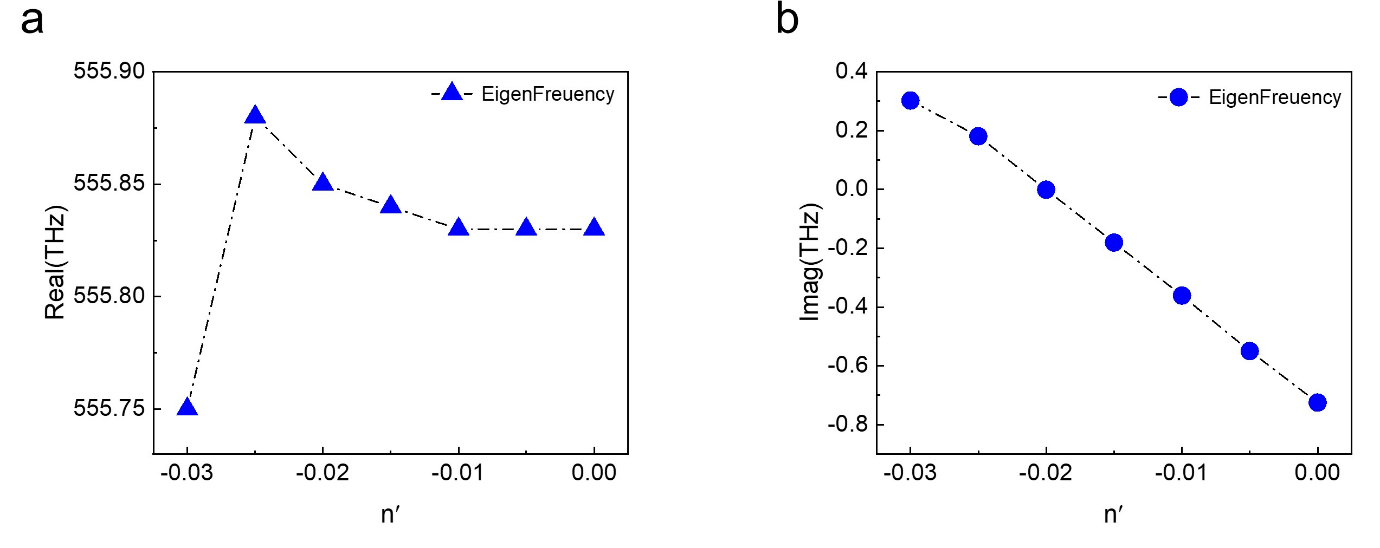


**Figure S3.** Quasi-BIC microlasers in BIC metasurface. (**a**) and (**b**) show the real and imaginary parts of quasi-BIC mode as a function of n”.

Theoretically, the size of the pump spot primarily affects the Q-factor of the resonator, which in turn influences the leakage of photons within the cavity. To analyze this point more quantitatively, we consider calculating the Q-factor and the guided mode wave vector *k* corresponding to the quasi-BIC mode. To further understand how the finite array size effect can affect the coupling distance, theoretical simulation was performed to investigate the field distribution and Q factors when only a finite array size of *N* by *N* was involved. Theoretically, BIC is induced via collective resonance of infinite electric dipoles in nano-antennas with coherent phase and intensity. When the laser spot can only excite a limited number of nano-antennas, this deteriorates the resonance integrity of BIC and leads to a finite Q factor. When the array size increases, the mode changes from diffusive to a well-confined electric field within nano-antennas, as shown in Fig. S4(a). Figure S4(b) and S4(c) illustrate the corresponding Q factor with different array sizes and the Q factor grows rapidly as an array size increases.


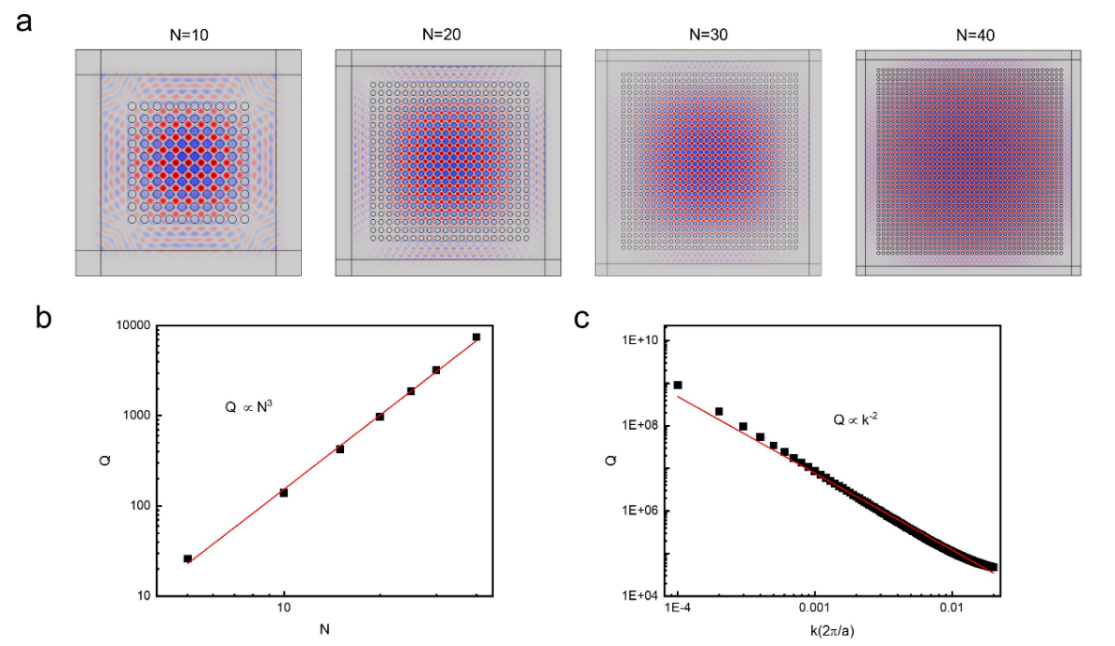


**Figure S4.** Simulation results explain the finite array size effect. (**a**) Electrical field distribution of BIC mode arranged with finite array size (*N* by *N*). (**b**) Calculated Q factors for the BIC mode with different array sizes, Q ∝ *N*^3^ (**c**) Q−*k* dispersions at infinite size, Q ∝ *k* ^-2^.

The fitting curve in Fig. S4(b) shows the Q factor is directly proportional to the cube of *N*(*N*^3^). This can be attributed to the coherence of electrical dipoles that forms Quasi-BIC with a reduction of field leakage to the free space. These simulation results agree well with the analytical theory reported in the literature^1^ ^2^ ^3^. On the other hand, as shown in Fig.S4(c), the Q factor of Γ-BIC decay quadratically with respect to *k* vector(Q(k) ∼ *k*^-2^)^4^. Therefore, the relationship between the *k*-vector and the finite array size N can be expressed as: *k* ∝ *N*^(-3/2)^. The ideal BIC mode can propagate to an infinite distance. As *N* increases and *k* decreases, the resonant mode approaches the ideal BIC mode. Therefore, the larger the *N*, the farther the propagation distance.

**BIC waveguide mediated long-range interaction**

In additional to the formation of numerous quasi-BIC microlasers, their mutual interactions are also essential for the realization of a photonic circuits. This characteristic relates to another intrinsic advantages of BIC metasurface. As depicted in Fig. S5, there are two types of leakages of the quasi-BIC microlasers. One is the well-known quasi-BIC mode in the normal direction. The other one is the propagation along the waveguide. While BIC degrades to quasi-BIC in the lasing area, its lasing wavelength is well preserved as the same as ideal BIC mode. In this sense, the propagating waves outside the pump area can propagate as a perfect waveguide mode without considering the material absorption and scattering loss.


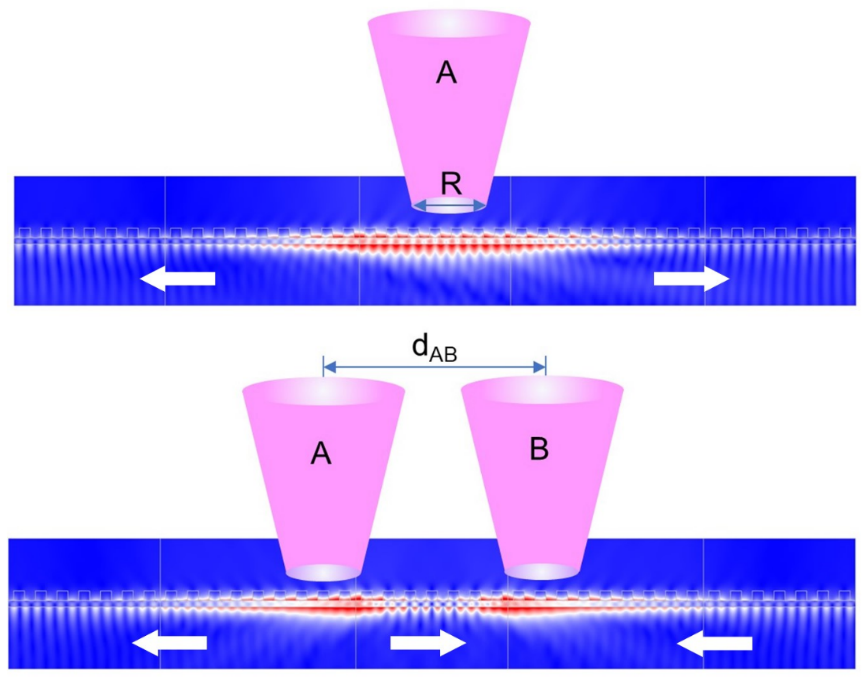


**Figure S5.** The field pattern of quasi-BIC microlaser. The arrows represent the directions of energy flow.

To demonstrate the long-range interaction mediated by the guide-mode in the BIC structure, we construct a two-dimensional structural model as shown in figure S6(a), where perfect matching layers are used on the top and bottom, and periodic boundary conditions are used on the left and right to solve for the eigenfrequencies. Figure S6(b) shows the electric field distribution of the structure under the condition of asymmetric dual-beam pumping, where the imaginary part of the refractive index of the pump region is set to n’’(A)=-0.02 and n’’(B)=0.02, respectively.


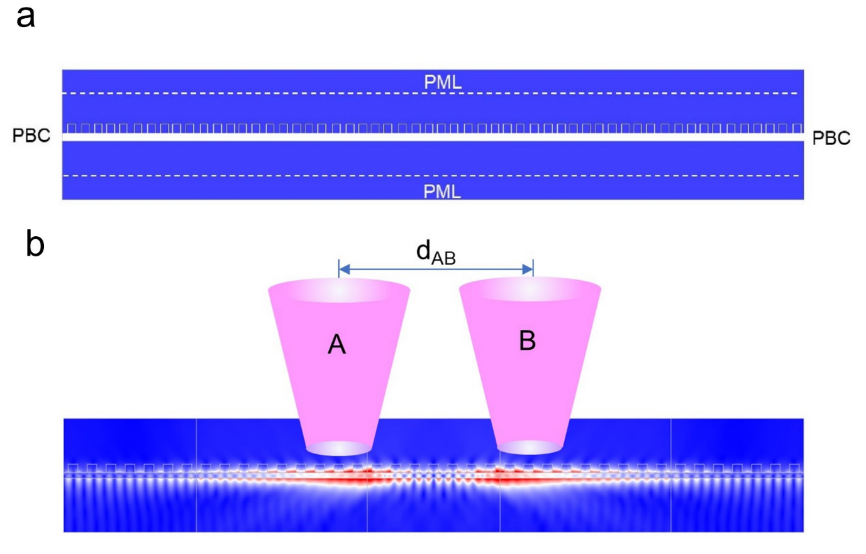


**Figure S6.** Numerical simulation for long-range interaction in the BIC structure. (a). Two-dimensional BIC structure model. (b). Electrical field distribution when two pumping beams asymmetrically pumped on the sample.

The distant strong coupling between quasi-BICs is completely different from traditional micro-disk cavities. As depicted in Fig. S7 (a), the whispering gallery modes are well trapped with total internal reflection in one cavity and only interact with the modes in the other cavity via the evanescent waves. Due to the exponential decay of evanescent waves, the coupling constant is usually below one or a half wavelength. Meanwhile, the loss factor of such confinements below light cone is typically quite small. It is not easy to construct the exceptional point (EP) below the critical line and the corresponding lasing self-termination phenomenon.

The case of quasi-BIC microlaser is totally different. BIC mode has zero radiation loss in the vertical direction and mostly resonant along Γ-M and Γ-X directions. This information can be seen in the near field image in the insets of Fig. 2(d) in main manuscript. As a result, around only a part of radiation reaches to another quasi-BIC resonator, and the other outgoing waves only contribute to optical loss and give a relatively large loss factor *k*. As shown in Fig. S7(b), it is straightforward to see that the loss factor should be around 3 times (or more) of the coupling factor.


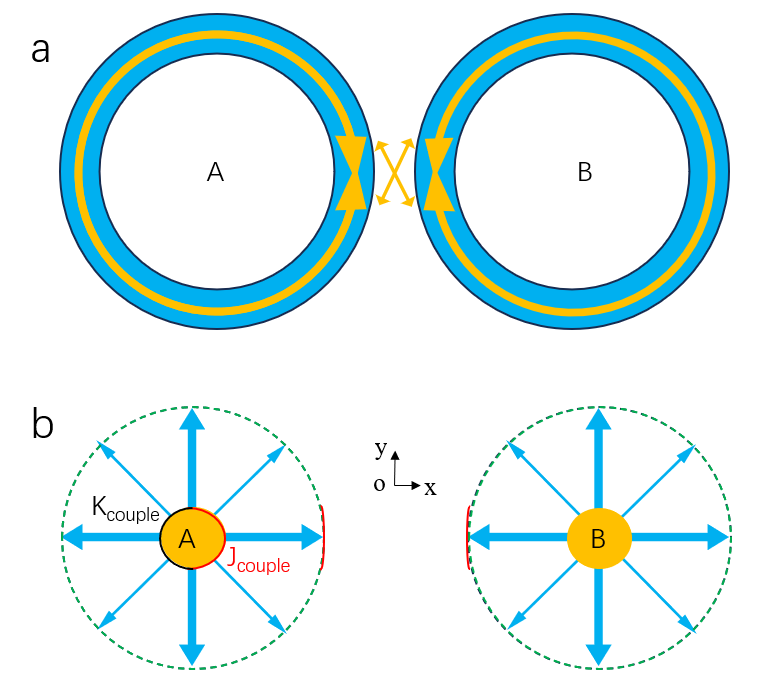


**Figure S7.** Schematics for the interaction between two microdisks (a) and two quasi-BIC microlasers (b). *J_couple_* represent the ratio of photons coupled with another quasi-BIC resonator, while *K_couple_* represent the ratio of photons propagate other direction that don’t couple with another quasi-BIC resonator.

## Note-2. Sample preparation

Here we show the detailed information of the fabrication of BIC metasurface. Since the lead halide perovskites are relatively instable in most solutions, we select the non-etching BIC and experimentally realize it with a conventional electron-beam lithography process. The details are summarized in Fig. S8 below. Before sample fabrication, the substrate, a 13 nm ITO coated glass substrate, is sequentially cleaned by ultrasonication in acetone, isopropanol and deionized water for 10 min each and then treated with ultraviolet light and ozone for 15 min. The precursor solutions of N_2_F_8_ ((NMA)_2_FA_n-1_PbBr_3n+1_, n=8) perovskite was achieved by adding a 25% molar ratio of 1-naphthylmethylamine bromide (NMABr) into a 1:1 ratio of HC(NH_2_)_2_Br (FABr) and PbBr_2_ in DMF at 0.4 M and stirred at 60 °C for 12 h. Then all the substrates and precursor solutions are moved to a nitrogen-filled glove box (H_2_O and O_2_ concentrations of <0.1 ppm). The Quasi-2D perovskite film was prepared using a one-step spin-coating method. Basically, the precursor solution is spin-coated on the ITO layer at 5,000 r.p.m. for 30 s. During the spin coating, 0.3 ml of ethyl acetate is dropped onto the perovskite precursor layer. The substrate is baked on a hotplate at 85 °C for 15 min and then the perovskite film is obtained. The thickness of the perovskite film is 100 nm, which is measured by a three-dimensional laser scanning microscope (Olympus LEXT OLS5100). Then 100 nm electron-beam resist (ZEP260A) is spin-coated onto the perovskite film and placed at room temperature overnight. The electron-beam resist is patterned with electron-beam aligner (Raith e-LINE, 30 kV). After developing in N50 for 60 s and dried with nitrogen gas, the exposed area is removed and the BIC metasurface can be finally obtained.


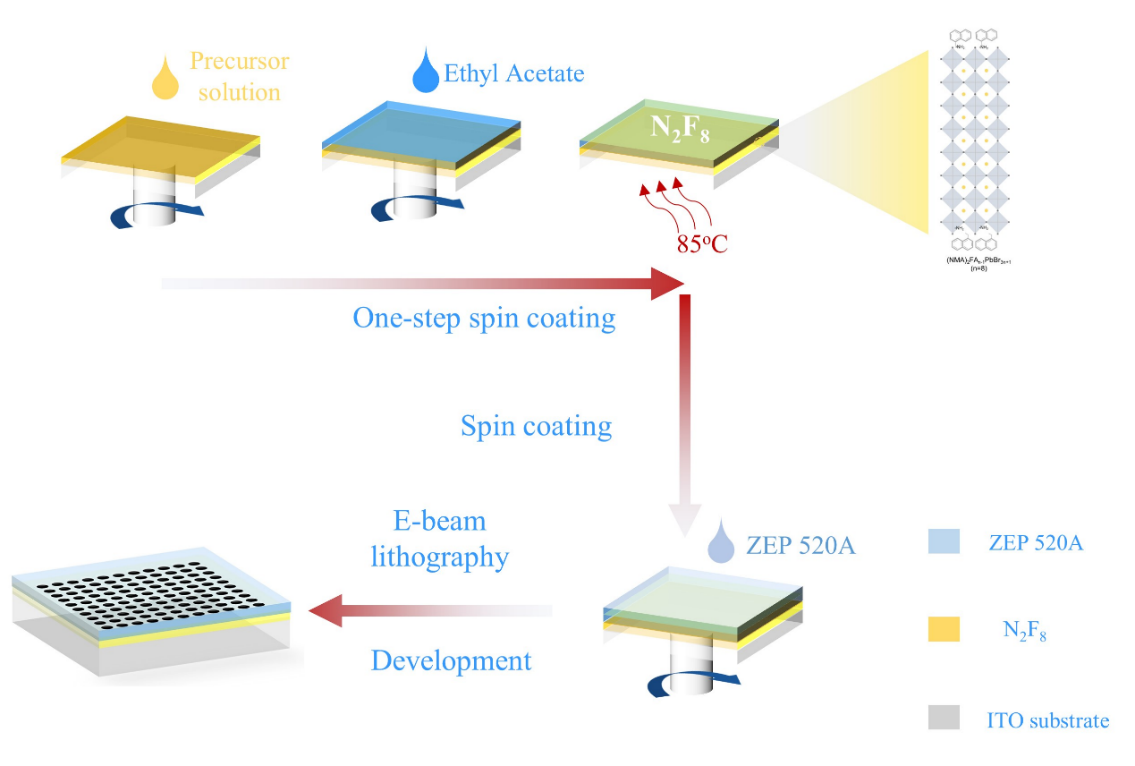


**Figure S8.** The fabrication process of the BIC metasurface. It is consisted of square latticed periodic holes in a ZEP520A film.

For the design of BIC metasurface, the refractive index (n) and the light extinction coefficient (k) play an essential role. After the synthesis of the quasi-2D perovskite film, its materials properties are then measured with ellipsometer. The fitted results are plotted in Fig. S9. The quasi-2D perovskite film has a relatively large refractive index (n > 2) over the entire visible spectrum and negatively small absorption below the band-edge (wavelength λ > 550 nm). The refractive index around the excitonic peaks of quasi-2D perovskites are increased to almost 2.4.


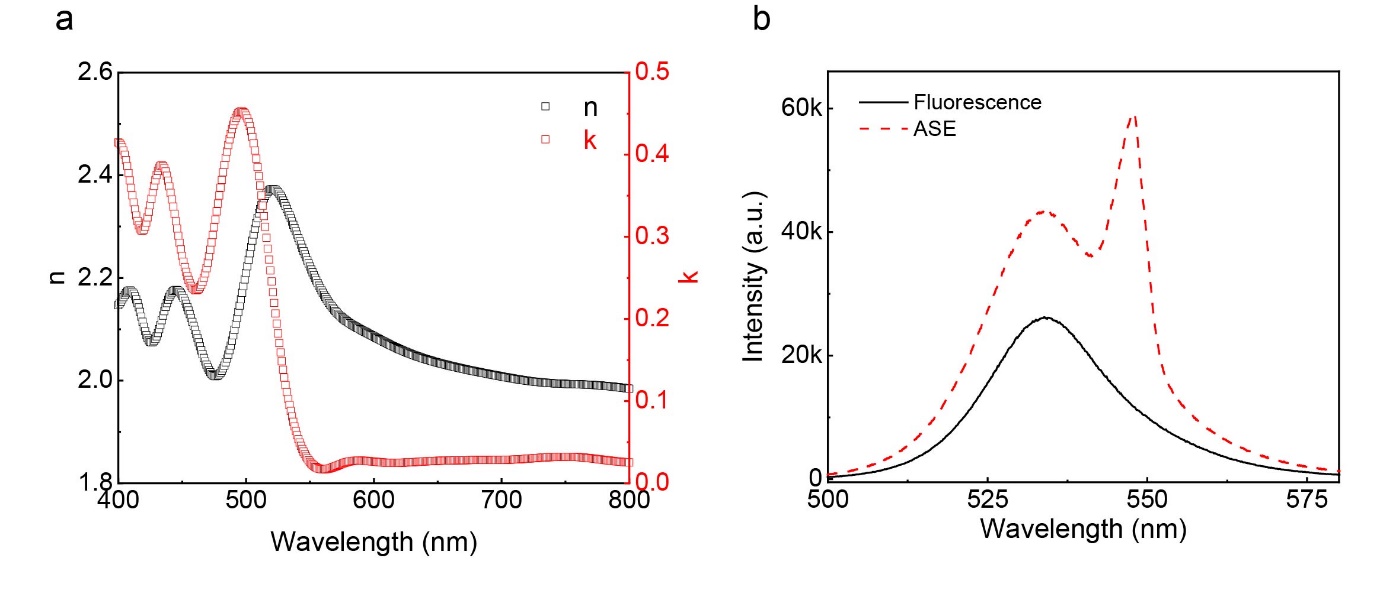


**Figure S9.** Optical properties of quasi-2D perovskite film. (**a**) Experimentally measured refractive index (n, blue curve) and light extinction coefficient (k, purple curve) of the synthesized quasi-2D perovskite film. The gain spectrum is highlighted by a vertical red shadow. (**b**) The recorded photoluminescence (solid line) and amplified stimulated emission (dashed line) of the quasi-2D perovskite film. All the experimental results are achieved at room temperature.

Then the emission characteristics of perovskite film are measured. Here the sample is excited by a frequency doubled femtosecond laser (400 nm, repetition rate 1 kHz, pulse width 100 fs). The incident laser is focused to a straight line on the sample by a cylindrical lens. When the pumping density is low, a broad emission peak centered at 532 nm can be clearly seen (solid line in Fig. S9(b)). The full width at half maximum (FWHM) is about 25 nm. All these results are consistent with the previous reports and show the spontaneous emission of the prepared quasi-2D perovskite film well. With the increase of pumping density, the gain the film is larger than the optical loss. As a result, the propagating waves in the perovskite film shall be amplified and the emission spectrum changes accordingly. The dashed line in Fig. S9(b) shows the corresponding spectrum of amplified stimulated emission (ASE). It is clear to see a narrow peak positioned at 547 nm with a FWHM of 6 nm. Thus, we know that our quasi-2D perovskite only has a quite narrow band gain spectral range. Such information is critical for the design of single-mode quasi-BIC microlasers.

## Note-3. Optical setup for lasing experiments

**Setup for conventional photoluminescence and lasing measurements**

Similar to the ASE experiment, a Ti:Sapphire femtosecond laser (Spectrum Physics) is used to excite the quasi-2D perovskite. The optical setup is shown in Fig. S10. The Ti:Sapphire laser is frequency doubled after passing a BBO nonlinear crystals. The unconverted components are filtered out by a narrow band filter. Then the laser beam is focused by a 20X objective lens onto the sample. To accurately determine the pumping position, a white light source (Thorlabs SLS201L/M) is added to the optical setup via a 50:50 beam splitter. The emission from the sample is collected the second 20X objective lens to a collimated beam. The collimated beam is divided by a 50:50 beam splitter after passing a long-pass filter. The reflected one is focused by an optical lens and analyzed by a spectrometer. The transmitted one is imaged by a 4-f system to monitor the back focal plane image of the objective lens with a CMOS camera.


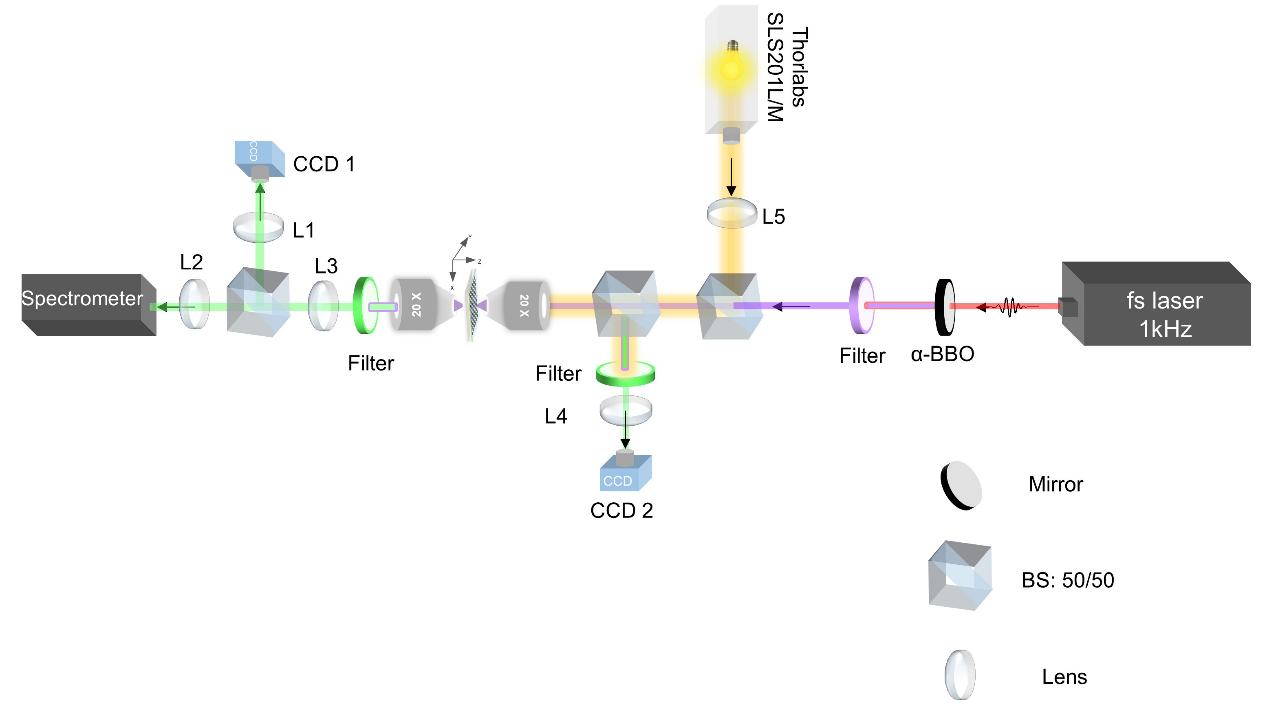


**Figure S10.** The optical setup for conventional lasing experiment. Both the emission spectra and far field patterns can be recorded.

With the above setup, we have characterized the emission spectra of the quasi-BIC microlaser. The experimental results are summarized in Fig. S11. Here the size of pumping beam is fixed at the diameter of D = 5 μm. When the pumping power is low, there is only a broad photoluminescence peak. Once the pumping density is above 25 μJ cm^−2^, a sharp peak emerges and dominates the emission spectrum. Figure S11(a) shows the integrated output intensity as a function of pumping density. A clear “S” shaped curve can be seen, indicating the transitions from spontaneous emission to lasing emission and finally to gain saturation. Insets in Fig. S11(a) are the corresponding near-field and far-field images at pumping fluence above 25 μJ cm^−2^. Both bright spots and donut-shaped directional output can be observed. Therefore, the lasing actions from the finitely pumped BIC metasurface can be confirmed. Figure S11(b) shows the polarization characteristics of the far-field pattern. By placing a linear polarizer before the camera, the far-field pattern changes from a "donut" to two sidelobes. With the rotation of linear polarizer, we find that the direction of sidelobes is always parallel to the main axis direction of the polarizer. Then we know that the far field radiation of the BIC is radially polarized. All these observations are consistent with the momentum space structures of optical BIC well.


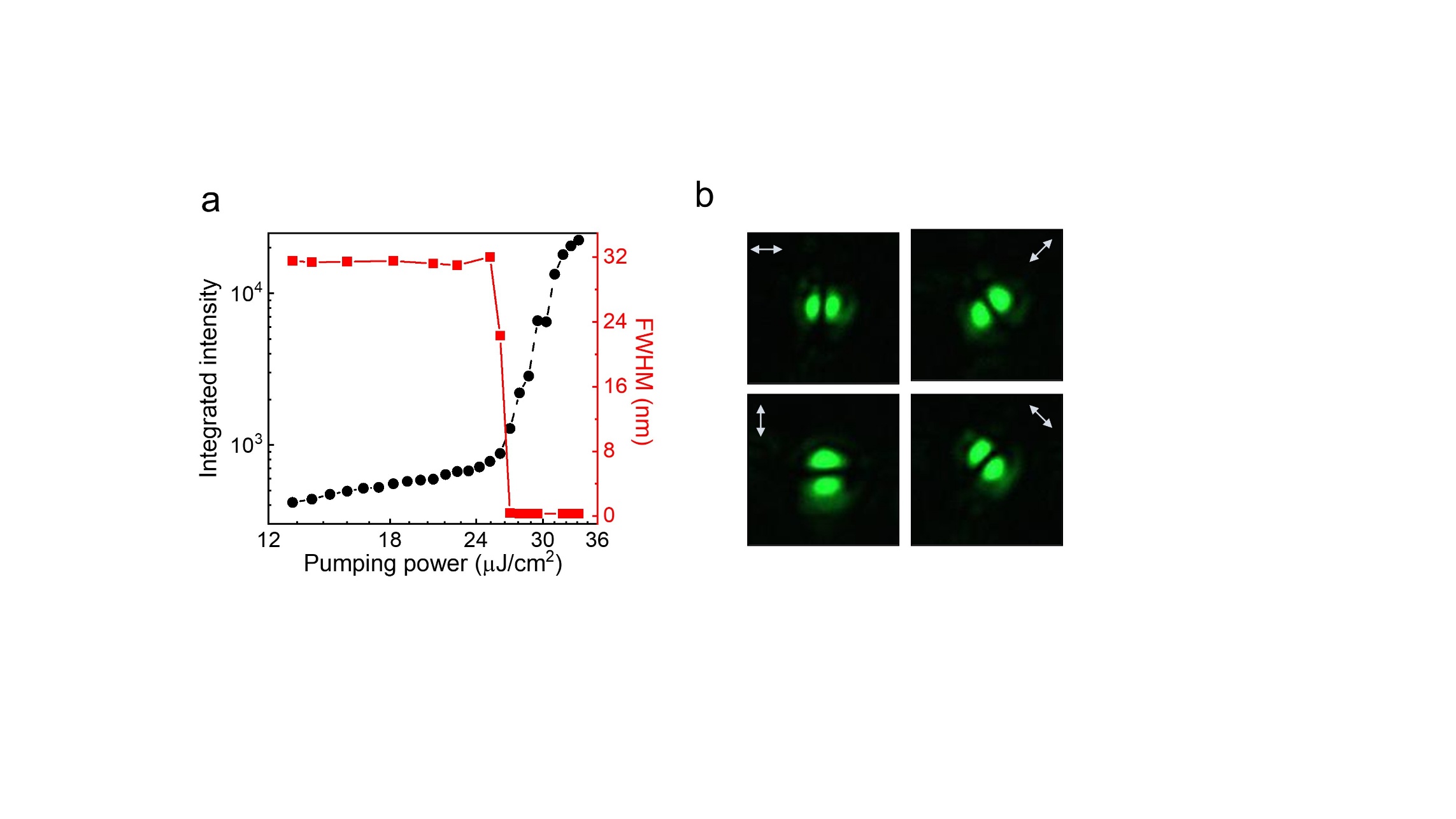


**Figure S11.** The characterization of a single quasi-BIC lasers. (**a**) The output intensity (purple dotted line) and the FWHM (green dotted line) as a function of pump fluence. (**b**) The polarization state of the donut shaped laser beam after a linear polarizer.

As mentioned in the main text, one advantage of the BIC metasurface is one metasurface can support numerous quasi-BIC microlasers with the nearly same emission wavelength. To confirm this characteristic, we have experimentally studied the repeatability and uniformity of quasi-BIC microlasers. As shown in inset of Fig. S12(a), we have measured the lasing spectra and laser threshold of quasi-BIC microlasers at five different positions named as A-E. The size of pump laser has a diameter. The corresponding experimental results are summarized in Fig. S10. It is obviously to see that the lasing peak is well maintained at the same wavelength ~ 544.5 nm. Both the lasing wavelength and the FWHM are almost identical at different positions (see Fig. S12(a)). By increasing the pumping power, the laser thresholds of five quasi-BIC microlasers have been recorded and shown as open squares in Fig. S12(b). The laser threshold also varies slightly around 25 μJ cm^−2^. All these observations clearly demonstrate the uniformity of quasi-BIC microlasers, essential for the study the distant coupling.


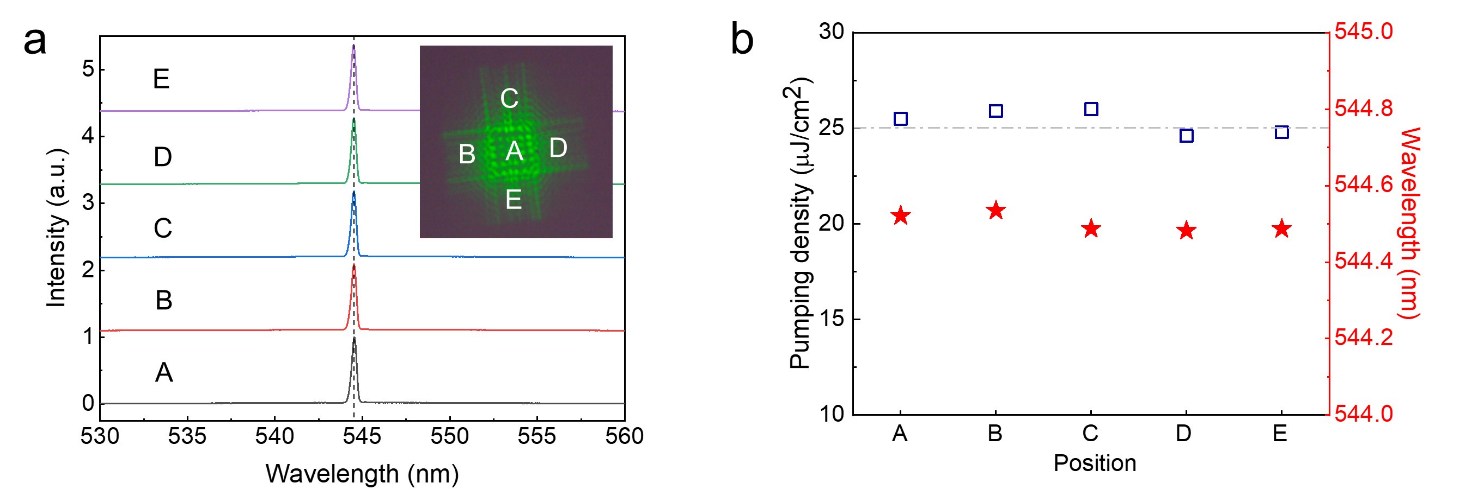


**Figure S12.** The unifomity of quasi-BIC microlasers in one meatasurface. (**a**) and (**b**) shows the emission wavelength and lasing threshold at different positions A-E in the inset.

**Optical setup for the distant interaction**

The uniformity of quasi-BIC microlasers at different positions and their intrinsic link via the lossless BIC mode make the quasi-BIC microlasers ideal for the study of distant interaction. The key information has been highlighted in the main text. Here we show the experimental details of the optical setup, which is shown in Fig. S13. The entire system is similar to a conventional pump-probe system. The pump beam at 400 nm from the Ti:Sapphire laser is divided into three beams by two 50:50 beam splitters. Two of them passes through two independent delay lines with controllable propagation distances. Neural density filters are applied to control powers of three laser beams. In the time domain experiment, the beam A that doesn’t experience the delay line determines the *zero* *point* in time. Two delays lines are tuned to control their delay times as the definitions in the main text. Three beams are combined together through beam splitters, and then focused onto different positions through the objective lens. The pumping positions of three beams are determined by the incident angle and locations on the objective lens. The relative time delay during the focusing process is compensated with the scanning galvanometers. The detailed information can be seen in the inset of Fig. S13. Similar to Fig. S10, white light source has also been introduced to accurately determine the locations on the sample. The emissions from the BIC metasurface are collected by another objective lens and analyzed by a spectrometer.


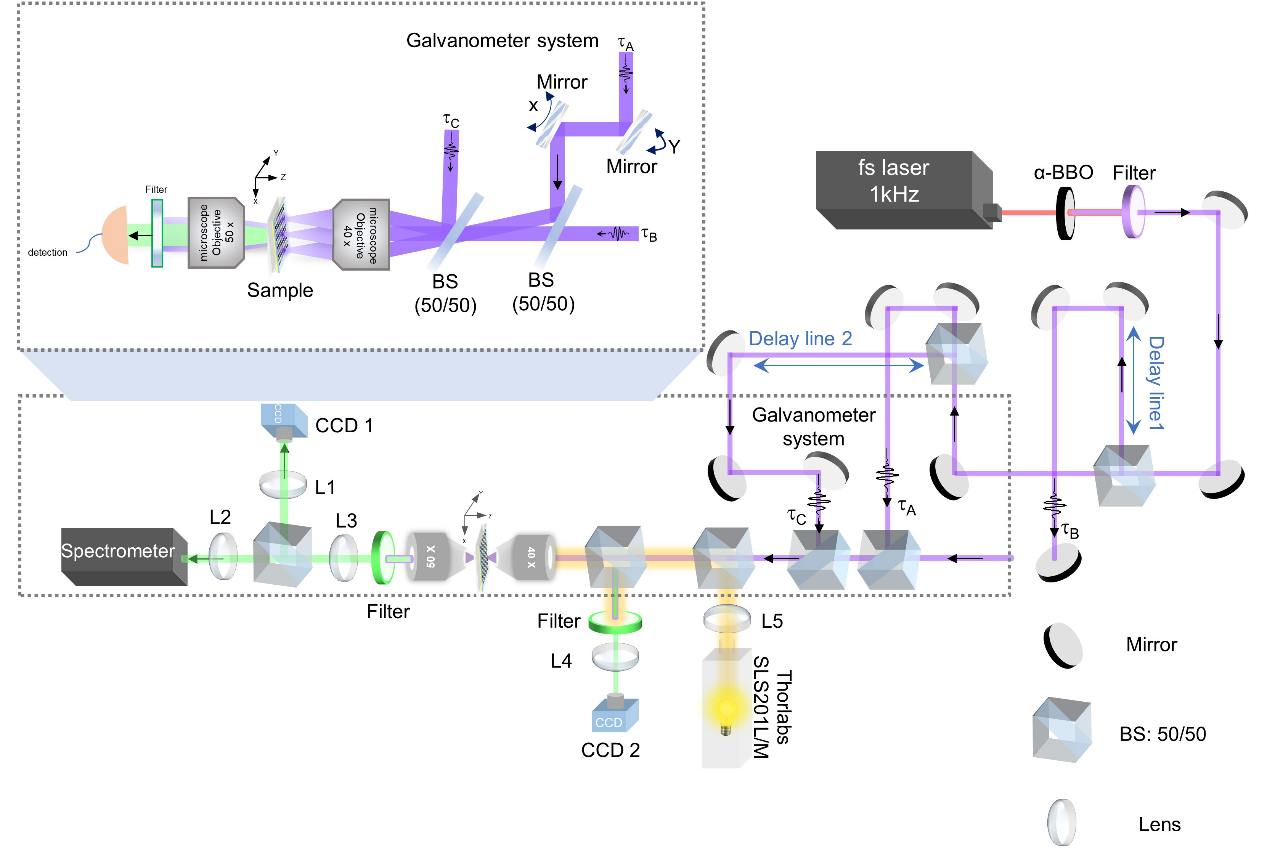


**Figure S13.** Optical setup for measuring distant interaction between multiple quasi-BIC microlasers. The 400 nm laser from the titanium sapphire laser is split into three beams by two 50:50 beam splitters. Two of the beams pass through independent controllable delay lines. A neural density filter is used to control the power of the three beams. The three beams are combined by the beam splitter and then focused to different positions by an objective lens. The pump positions of the three beams are determined by the angle of incidence and the position on the objective lens. The relative time delay during focusing is compensated by a scanning mirror. A white light source is also introduced to precisely determine the position on the sample. The light emitted by the sample is collected by another lens and analyzed by a spectrometer.

## Note-4. Long range interaction at BIC

Ideally, the absorption loss of BIC metasurface is ignored and the coupling constant *J* is kept as a constant and independent on the separation distance. Practically, the propagation of lasing mode in BIC metasurface shall experience two types of loss, i.e., the radiation loss and the material loss. The first one comes from the frequency shift due to the excitation (see main text). While the shift is relatively tiny, it deviates from the perfect BIC condition of the unpumped area and can only propagate as a quasi-BIC mode. The absorption loss is caused by the material absorption. This is because that a lasing material is also an absorptive material at the same wavelength. Then the coupling constant becomes inversely dependent on the separation distance *d*. According to the coupled mode theory, the interaction between two quasi-BIC microlasers can be tuned by controlling the separation distance *d*.

The dependence of coupling coefficient *J*(*d*) on the separation distance can be estimated experimentally. Figure S14 shows the experimental setup. Basically, one quasi-BIC microlaser is excited by focusing 400 nm femtosecond laser onto the BIC metasurface with a 20X objective lens (top one, NA= 0.2). The bottom objective lens (20X, NA=0.2) is applied to collect the emissions at different distance away from the pumped area. When the excitation density is above threshold, the quasi-BIC microlaser leaks mainly Γ-M and Γ-X directions. The propagation mode will be absorbed by the gain material and partially scattered by the top gratings. In this sense, laser intensity collected by the second objective lens at different positions should be different too and can reflect the trend of coupling constant. One example is illustrated in Fig. S14(b). Here the excitation density at objective lens-1 is fixed at 1.2 P_th_. The pumping area and the collection area are both fixed with a beam diameter around D = 10 μm. The integrated intensity of lasing mode is recorded and shown in Fig. S12(b) as a function of distance *d*. With the increase of separation distance, we can clearly see the slightly reduction of integrated intensity.

To more accurately analyze the influence of spot size on the coupling distance, we measured the intensity of the collected spectrum under different pump spot sizes, as shown in the Fig. S14(c) below. It can be observed that the larger the spot size, the slower the spectral integrated intensity decays with increasing distance. Therefore, it can be concluded that the larger the spot size, the longer the coupling distance between the quasi-BIC resonators.

**
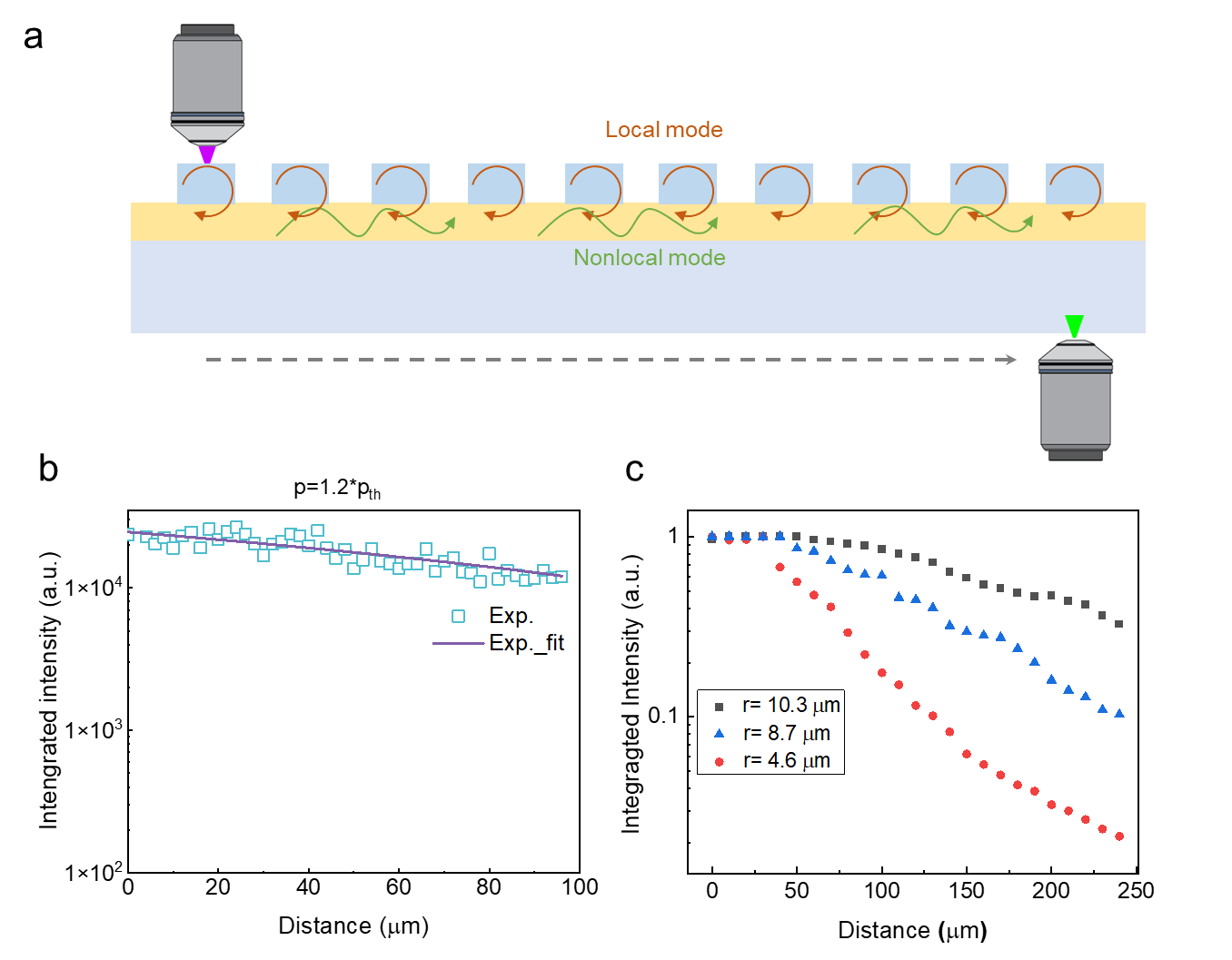
**

**Figure S14.** Propagation in BIC waveguide. (**a**) Schematic of optical setup for characterizing the emission intensity at different separation distance. (**b**) The experimental results and the corresponding exponential fitting results. (**c**) Normalized integrated emission intensity under different pump spot sizes with the horizontal distance changed.

The reduction of collected laser intensity at large d can be fitted with an exponential decay curve. The corresponding trend is then applied to the theoretical model and the numerical results are shown in Figs. S15. The coupling constant is dependent on the position following the equation $J=5.0\exp\left( {-d}/D \right)$, where *D*=190 μm is fitted from Fig. S14(b), and the loss factor is dependent on *J* ($k=(16.3-\alpha*d-J)$), where $\alpha$ is the absorption coefficient of materials. Then the coupling constant and loss factor are dependent on the separation distance. It is easy to see that two modes weakly interact with each other at the and maintains at the same frequency when the separation distance is above 40 μm. With the decrease of separation distance, the coupling gets stronger and the loss factor is smaller. As a result, the interaction increases and the coupled systems approach the exceptional point. Since the exceptional point is below threshold, a region of lasing self-termination is thus generated, and it can be seen lasing self-termination generated when 30 μm < *D_AB_* < 45 μm. All these numerical results are consistent with the experimental observations in the main text very well. Base on the above experimental results and theoretical analysis, we know that the real BIC metasurface can control the distant coupling between two quasi-BIC microlasers via the separation distance.


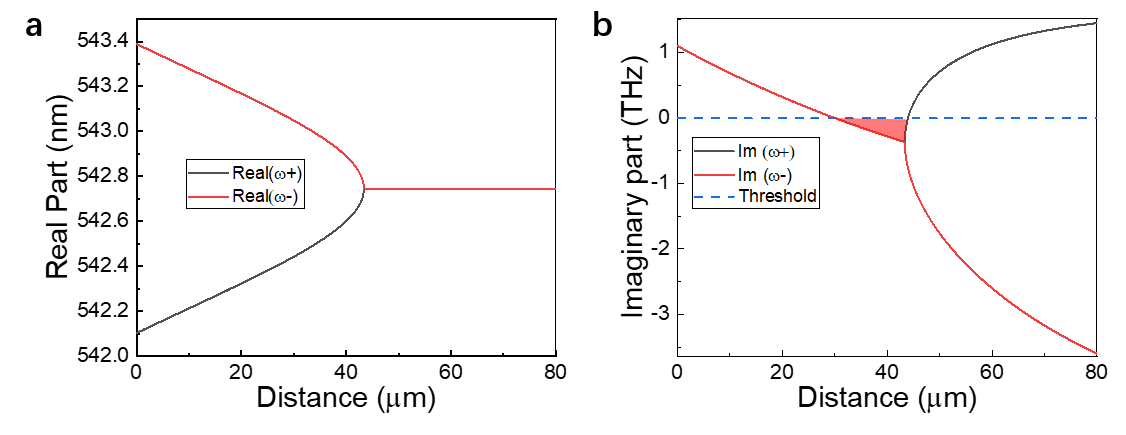


**Figure S15.** Theoretical fitting of the experimental observations in Fig. 3(b) of the main text. Here the fitting parameters are set as $\mathrm{Re}\left( \omega_{0} \right)=3473 \mathrm{THz}, \gamma_{a}=16 \mathrm{THz}, \gamma_{b}=8.1 \mathrm{THz}$, $J=5.0\exp\left( {-d}/D \right)$ THz, where *D* = 190 μm

We also measured the emission spectrum for intensities of beam A and B are both lower than the laser threshold, e.g. power of both beams are set as 0.85 P_th_. As shown below, only spontaneous emission spectrum appeared when there is only a single beam pumping the sample. When two beams are pumped simultaneously, there are two situations. One is when the distance between the two beams is relatively close, such as 16.12 μm We see an enhancement in the luminescence spectrum and a split double peak, indicating coupling between the two regions. Another situation is that when two beams are far apart, such as 36.51 μm, the emission spectrum also increases, but only fluorescence appears, indicating that there is no coupling between the two regions. This may be because although the pump region is close to the laser threshold, the gain of a quasi-BIC resonant cavity still cannot overcome the loss to generate lasing emission, so the fluorescence generated by the quasi-BIC resonant cavity cannot propagate further in the plane. When two quasi-BIC resonators approach each other, the fluorescence generated by quasi-BIC resonance can gain each other, thereby generating lasing emission and forming a coupling system.


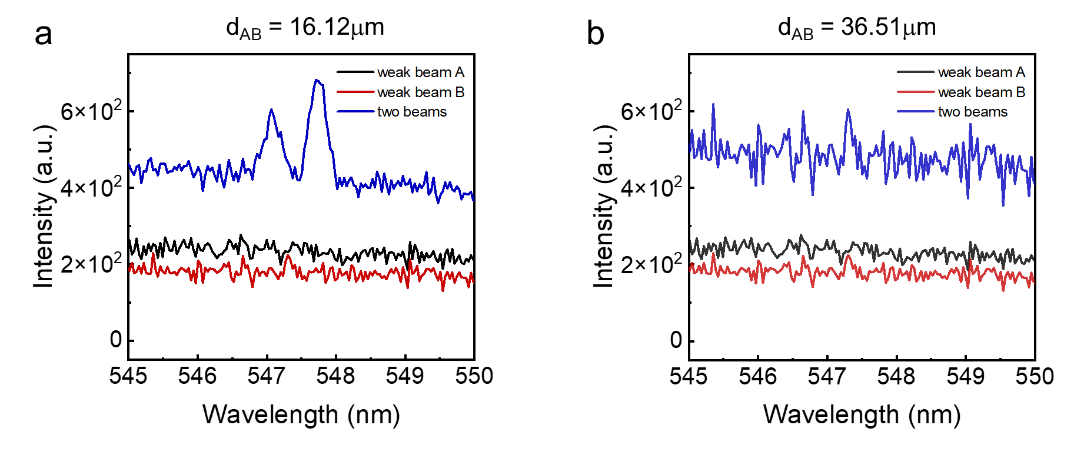


**Figure S16.** Long-rang interaction for pumping power below lasing threshold. (**a**) Emission spectrum for two beams set apart as 16.12 μm. (**b**) Emission spectrum for two beams set apart as 36.51 μm.

The distance-dependent coupling between quasi-BIC cavities is indeed a crucial aspect of our study. Here, we provide more detailed information. As shown in the Fig. S17 below, the diameter of our pump spots in the experiment is fixed at 5 μm, and the distance *d* between the two resonant cavities is defined as the length between the two centers. It can be seen that when it is less than 10 μm, the emission spectrum of the sample presents a single laser emission peak, but compared to the uncoupled case (i.e. when the two beams are quite far apart, as shown in the spectrum at *d* = 70.15 μm in the figure), the laser emission peak is significantly enhanced and exhibits a blue shift. This situation usually occurs when a single laser saturates its gain, which means that the two resonant regions function as a unified resonant cavity. As the distance gradually increases and exceeds 10 μm, a split dual lasing emission peak can be observed in the emission spectrum, indicating that the system has become a coupled system composed of two independent resonant cavities. So a simple conclusion can be drawn: when the distance between two pump beams exceeds the sum of their radii, the system can be considered as two independent quasi-BIC resonators.

**
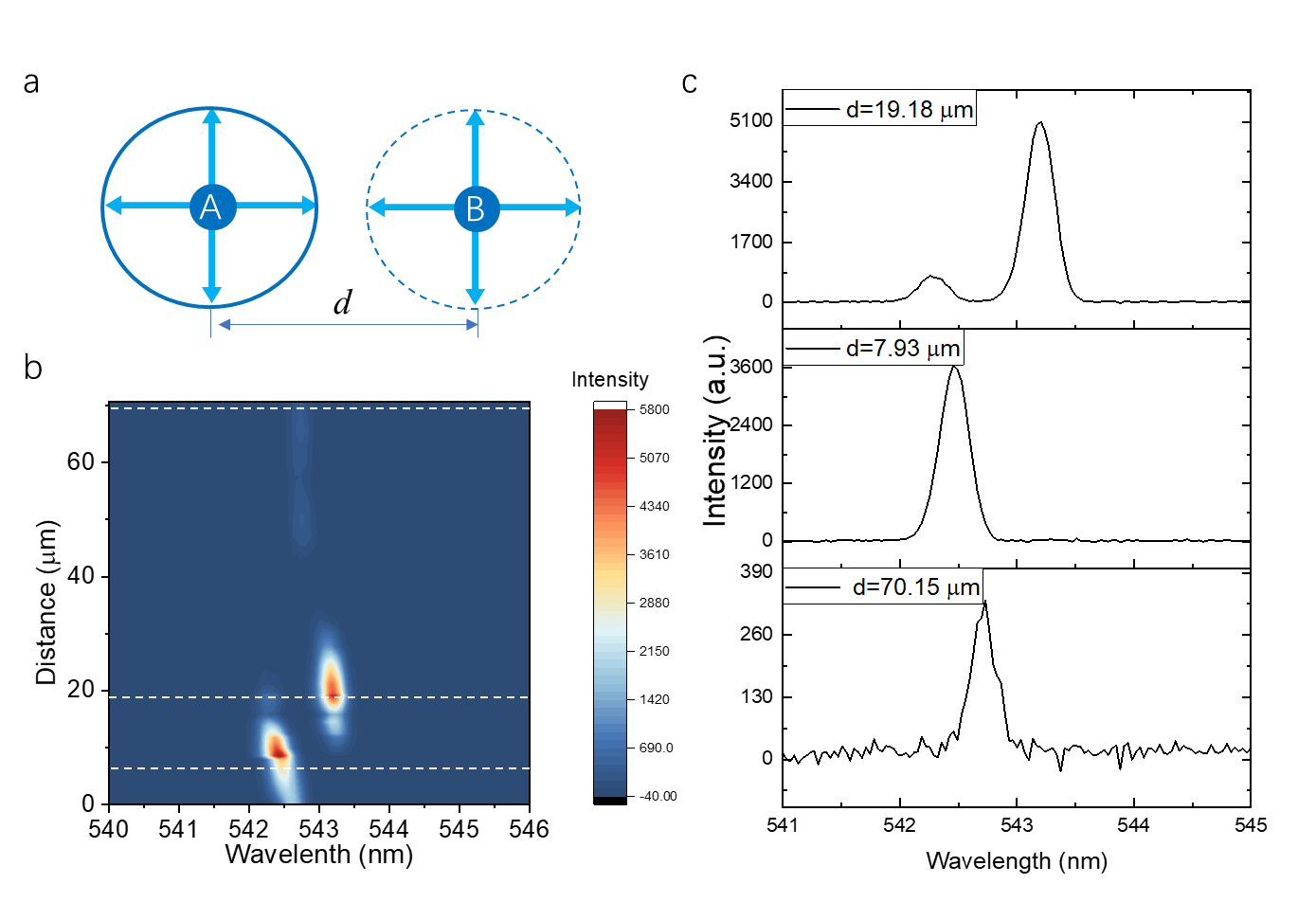
**

**Figure S17**. (**a**) Schematic diagram of two distant coupled quasi-BIC resonators, the distance *d* between two cavities is defined as the length between two centers of the circles. (**b**)Two-dimensional spectral map showing emission as a function of beam separation *d*. (**c**) Emission spectra at selected distances *d* = 7.93 μm, 19.18 μm, 70.15 μm, respectively.

## Note-5. Dynamic control of distant coupling

**Time-domain control**

The coupled mode theory also shows the dependence of eigenvalues on gain coefficients. In conventional studies, the gain coefficients are usually changed by the pump fluences. In the main text, we reveal that the time delay can be used to control the difference in gain coefficients between two quasi-BIC microlasers. As a result, the switch from single-mode laser to lasing self-termination and mode splitting has been realized within a time of 10 ps. Here we show the dependence of gain coefficients on the time.


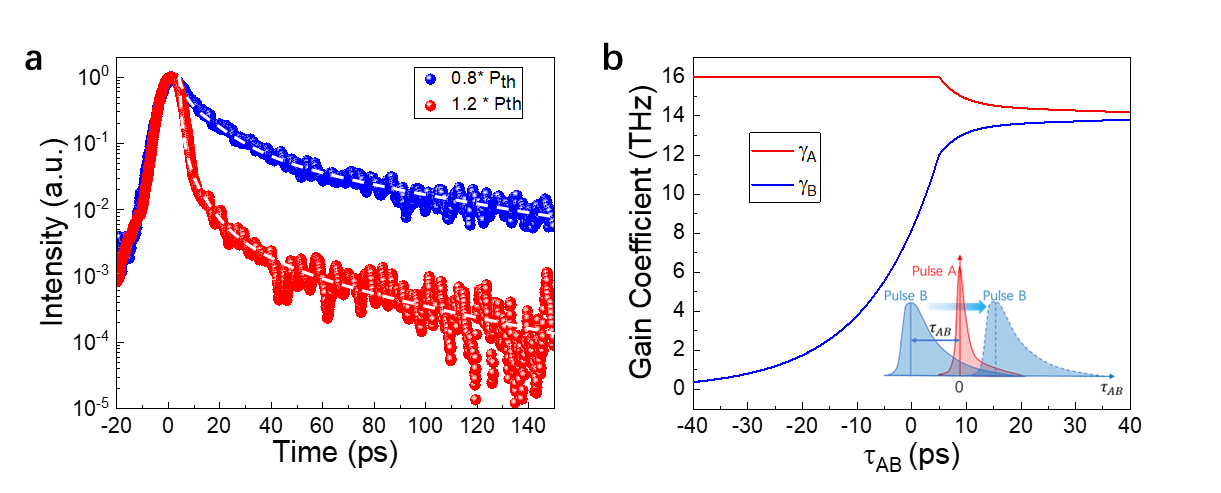



**Figure S18.** The experimentally recorded lifetime at different pump fluences. (a). Photoluminescence decay profiles(dots) of the BIC metasurface, showing the evolution from SE to lasing emission with increasing the pumping power. The white dashed line shows the fitted lifetime curve. **(b)**. Gain coefficients curve used in the numerically coupled mode model as a function time delay between A and B. here the fitting parameters are set as: $\tau$_a1_ =7 ps, $\tau$_a2_ =1000 ps, $\tau$_b_ =30 ps, $g_{a}$= 16 THz, $g_{b}$=10 THz.

Once ultrafast excitation and the corresponding relaxation are considered, it is easy to know that the population inversion keeps changing with the time. In this sense, the gain coefficient $\gamma_{a}$ and $\gamma_{a}$ are strongly time dependent. To achieve such time-dependence, we measured the lasing lifetime using the streak camera. Figure S18(a) shows the experimentally recorded results, and the white dashed line shows the fitted lifetime curve. It can be seen the photoluminescence intensity of the BIC metasurface decay rapidly after excitation when the pumping power is larger than the lasing threshold, showing the evolution from SE to lasing emission. In this sense, the gain coefficient can be expressed as a function of time, For pumping power is 0.8 P_th_, $\gamma_{b}=g_{b}*\exp\left( {-t}/{\tau_{b}} \right)$, for pumping power is 1.2 P_th_, $\gamma_{a}=g_{a1}*\exp\left( {-t}/{\tau_{a1}} \right)+g_{a2}*\exp\left( {-t}/{\tau_{a2}} \right)$. Here $\tau$_a(b)_ are the fitted results and $g_{a(b)}$ are the factor of maximal gain. From this equation, it is easy to know that the gain coefficient is a function of time and the difference in gain coefficients between two beams can be tuned by the delay time. As shown in the inset of Fig. 14(b), we depicted the scenario of interaction between two quasi-BIC resonators when exciting the sample with two pumping beams separated by a certain time interval. When the B beam pumps earlier than the A beam (i.e., *Δt_AB_* < 0), the gain coefficient γb for the interaction of B with A increases with the time delay approaches to zero, while the gain coefficient for A remains constant. When the A beam pumps earlier than the B beam, the coupling will transfer gain from the more highly pumped spot to the other. Figure S18(b) shows the gain coefficients of the two beams as a function of the time delay.

Based on the numerically fitted time dependence, we have calculated the gain coefficients as a function of time delay and achieved the eigenfrequencies following coupled mode theory. All the results are summarized in Fig. S19. The entire system and process should be extremely complicated. For simplicity, we only consider that the gain coefficient as shown in Fig. S19(b). By taking this trend into the coupled mode theory, the fitted results can be clearly seen in Fig. S19. With the increase of delay time, we are able to see the transition from a nearly single mode lasing to lasing self-termination and eventually to the mode splitting. The lasing self-termination generated when -5 ps < $\Delta t_{AB}$ <5 ps. All these results are consistent with the experimental observation very well.


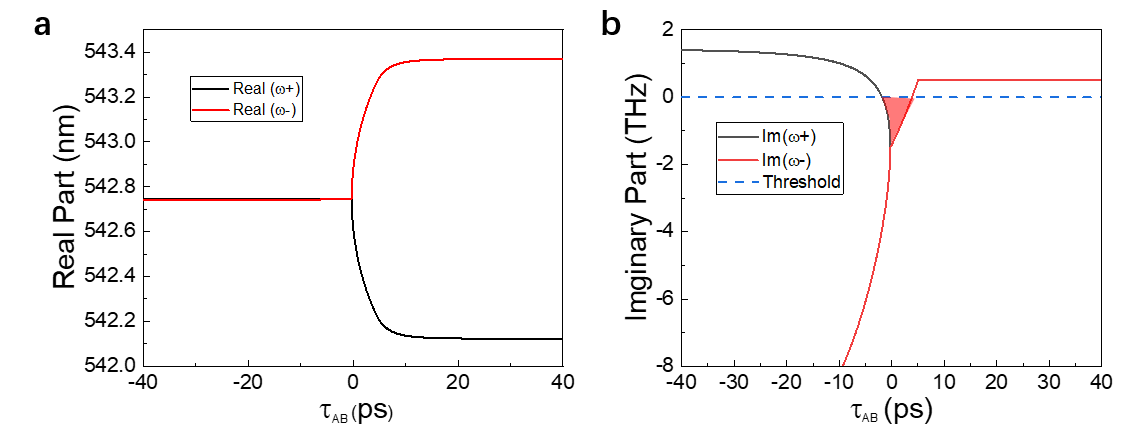


**Figure S19.** Theoretically calculated eigenvalues at different delay time. The real parts **(a)** and imaginary parts **(b)** of the eigenvalues. Here the fitting parameters are set as: $\mathrm{Re}\left( \omega_{0} \right)=3473 \mathrm{THz},$ $J=4 \mathrm{THz}$,$k=14 \mathrm{THz}$, and $\tau$_a1_ =7 ps, $\tau$_a2_ =1000 ps, $\tau$_b_ =30 ps, $g_{a}$= 16 THz, $g_{b}$=10 THz.

**Approaching to the EP point**

Due to the relatively large power difference between the two beams, we note the exceptional point (EP) was submerged within the lasing self-termination region. In fact, the virtual EP in the PT-symmetric system can be approached by adjusting the relative power difference between the two beams. Figure S20 (a) shows the theoretical mode calculations when there is zero frequency detuning between two resonators. When the two resonators are coupled, the mode becomes delocalized between the two disks, and the threshold lines are no longer parallel to the pump axes. When the pump powers of the two disks approach the lasing threshold, the coupling transfers gain from the more strongly pumped disk to the other disk. As a result, the threshold of the more strongly pumped disk increases compared to the uncoupled case, while the threshold of the less strongly pumped disk decreases, causing the threshold lines to approach each other (white dashed line). In the zero-detuning system, the threshold lines combine into a segment called the parity-time symmetric (PT-symmetric) line. The ends of the PT-symmetric line are the exceptional points (EPs) of the zero-detuning system, where the eigenmodes coalesce. Frequency detuning prevents this coalescence and creates an avoided crossing near the zero-detuning exceptional points.

The virtual EP in the PT-symmetric system can be approached by adjusting the relative power difference between the two beams. We conducted experiments by scanning the power of Beam B while maintaining Beam A at different power levels (always above the threshold) and measuring the output spectra. The experimental results are summarized in Fig. S20(b). It can be observed that as the power of Beam A gradually increases, the lasing gap progressively narrows. Figure S20(c) summarized the lasing gap under increased *P_A_*. When the lasing gap closes, a virtual EP is almost reached.


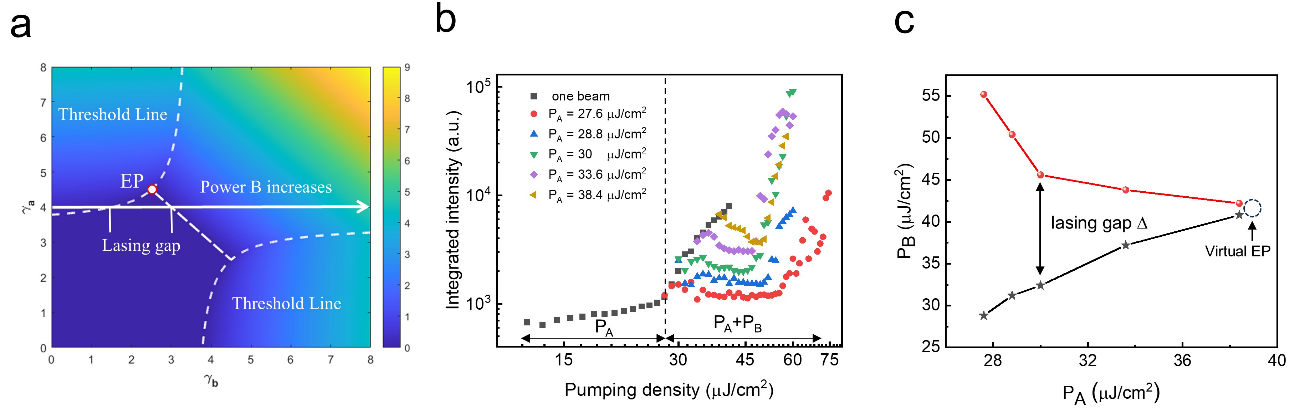


**Figure S20**. (**a**). Theoretical mode landscape of two coupled quasi-BICs with zero detuning. (**b**) Measured spectrum of two coupled quasi-BICs with only pumping power of beam B increases. In experiments, the pumping power of beam A is fix at 27.6 μJ cm^-2^. (**c**) Summarized lasing gap under increased *P_A_*. When the lasing gap closes, a “virtual” EP is almost reached.

Additionally, based on the experimental conditions of Fig. 3(b) in the manuscript, adjusting the relative power of the two beams can also bring the system close to the EP. As we previously discussed, changing the time delay corresponding to the changing of actual pumping power. Therefore, this scenario is essentially similar to above. Figures S21(a-d) display the emission spectra under varying time delays for different power differences between the two beams. It can be seen that as the power difference decreases, the manifestation of the lasing gap in the time delay domain also diminishes and nearly disappears when approaching the EP.


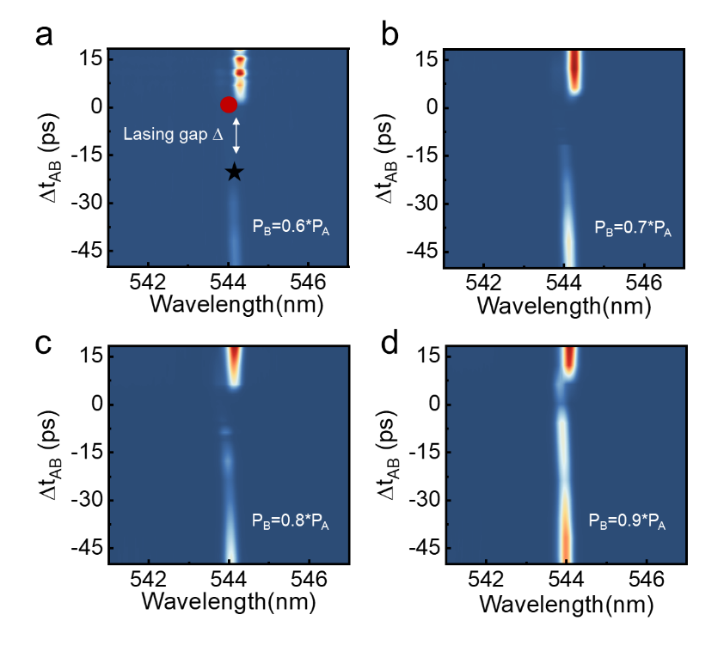


**Figure S21**. Emission spectra under varying time delays for different power differences between the two beams.

## Note-6. Zero mode in the three coupled quasi-BICs

As described in the manuscript, the BIC metasurface supplied a platform for multimode coupling, and we showed two examples for the three coupled quasi-BIC resonators. Here we show the theoretical model for the three-mode interactions. The non-Hermitian Hamiltonian of three-state interaction can be expressed as:

$H=\left( \begin{matrix} \omega_{0}+i(\gamma_{a}-k_{a}) & J_{ab} & J_{ac} \\ J_{ba} & \omega_{0}+i(\gamma_{b}-k_{b}) & J_{bc} \\ J_{ca} & J_{cb} & \omega_{0}+i(\gamma_{c}-k_{c}) \end{matrix} \right)$

where *γ* and *k* are the gain coefficient and loss factor of each mode. The coupling factor between different modes are *J_ij_* (*i, j=a,b,c; i*≠*j*). Similar to the above discussion, the coupling factor and the gain coefficient of the three modes can be modified by controlling the pumping power, time delay and separation distances. The eigenvalues $\omega_{n}$ of the non-Hermitian Hamiltonian is calculated using the modified Cardano method. When beam C doesn’t interact with the other two beams, i.e., $\gamma_{c}=0, k_{c}=0$ and $J_{ac}=J_{ca}{=J}_{cb}{=J}_{bc}=0$, the eigenvalues are calculated and shown in Fig. S22 as a function of $\gamma_{b}$. All the other settings are the same as two mode interaction. With the increase of $\gamma_{b}$, we can see that all the behaviors are exactly the same as two-mode interactions. Both the lasing self-termination region and the mode splitting regions can be clearly seen.


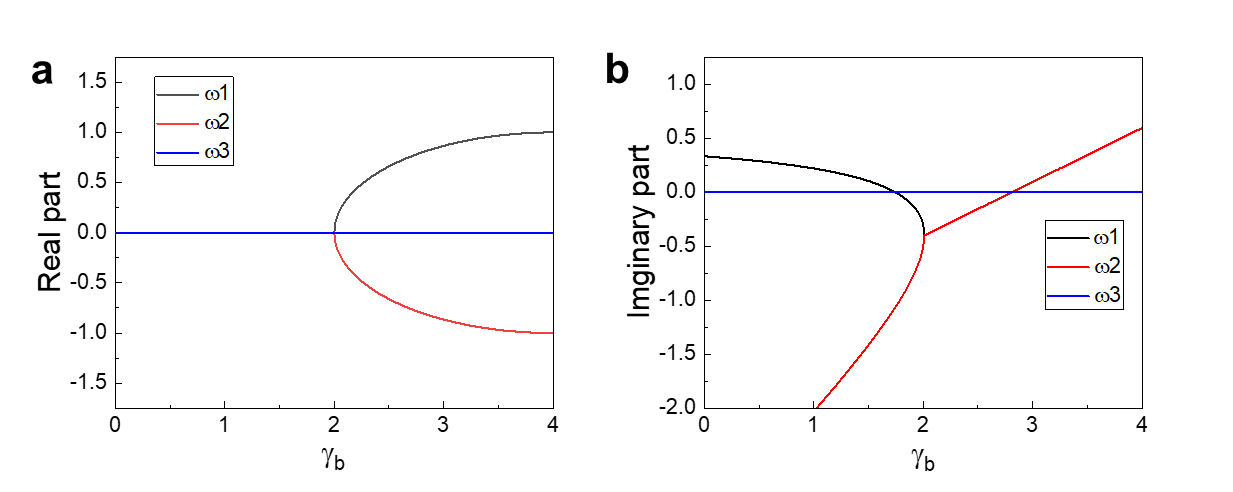


**Figure S22.** Eigenstate for three coupled quasi-BIC resonators. The third beam doesn’t interact with the other two and the entire system is the same as two-site interaction in the main text.

When beam C interact with the other two modes, we first consider the situation when beam C locate in the middle between A and B. In this case, the condition: *k_c_* < *k_a_* = *k_b_*, $J_{ab}=J_{cb}=0$ should be satisfied, since beam C locate in the middle between A and B, the coupling between A and B is interrupt, and the loss of site-C is smaller than A and B.Then we set $J_{ac}=J_{ca}{=J}_{cb}{=J}_{bc}=1$, $k_{a}=k_{b}=3.4$,$k_{c}=2.6$, $\gamma_{a}=\gamma_{b}=3.8$. Fig. S23 shown the eigenvalues as a function of $\gamma_{c}$. With the increase of $\gamma_{b}$, it can be seen that when $\gamma_{b}$ is relatively large, the real part of the eigenvalue splits into three values, and the imaginary part of the eigenvalue is all greater than 0, corresponding to the three-mode lasing when three beams are pumped simultaneously. When $\gamma_{b}$ < 2.2 ($\gamma_{b}$ < *k_c_*, below the threshold), only the imaginary part of mode 2 exceeds 0, and the frequency of mode 2 situates between 1 and 3, indicating that the system is in the zero-mode laser region.

**
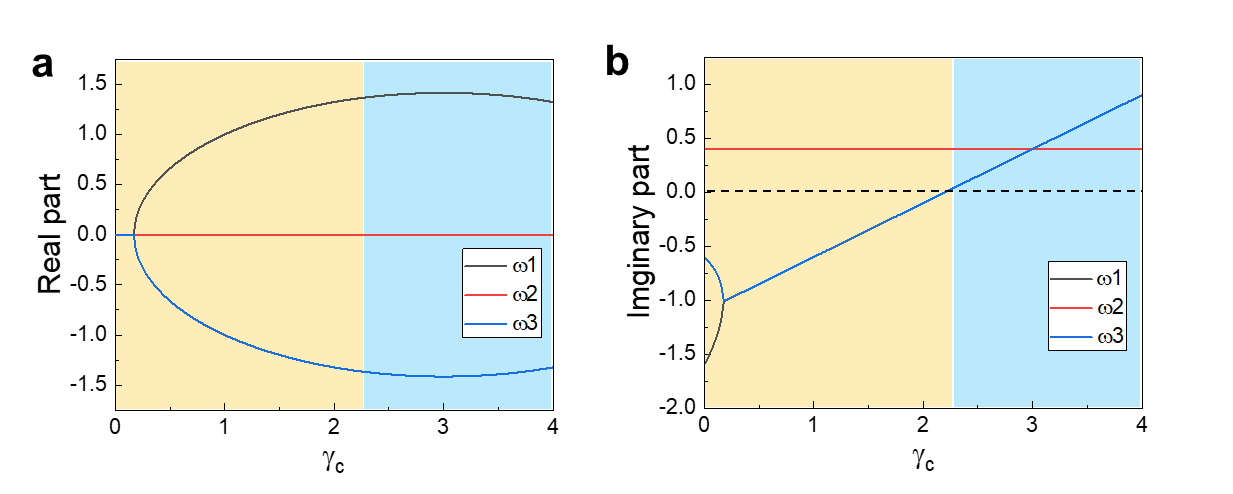
**

**Figure S23.** Zero mode formed by three coupled quasi-BIC resonators. (**a**) and (**b**) show the real and imaginary part of the three-body coupling system, respectively. Where the calculation parameters are set as: $J_{ab}=J_{cb}=0, J_{ac}=J_{ca}{=J}_{cb}{=J}_{bc}=1,$ $k_{a}=k_{b}=3.4, k_{c}=2.6$, $\gamma_{a}=\gamma_{b}=3.8$.

The zero-mode lasing can also be confirmed by the far-field patterns. The exact phase shift between adjacent cavities distinguishes the wave function of a zero mode from all other modes and dramatically impacts the PL far-field imaging in experiments, as shown in figure S24(a, b). We call ω_1_ and ω_3_ the highest and lowest frequency modes, respectively, both having a nearly symmetric field distribution, while ω_2_ is the central mode featuring π/2 phase jumps between adjacent cavities. Figure S24(a) shows the near field pattern of the eigenstates for the three coupled quasi-BICs, with each quasi-BIC cavity oscillating intensity distribution assumed to be a Gaussian function. The vertical white dashed lines in the near field indicate the position of the cavities, and figure S24 (b) shows the corresponding far field pattern. In the main text we have described the emission spectrum evolution as a function of time delay *Δt_AC_*_._ In Figure S24(c) and S24(d), we show the typical far-field pattern and correspondingly emission spectrum. When *Δt_AC_*=-17.4 ps, e.g. beam C pumps earlier than A and B, the gain of beam C decreased when the other two beams pump the sample, consequently, only a single lasing peak locate in the original position as a single beam pumps the sample, indicating the formation of zero mode. The corresponding far-field pattern *Δt_AC_* = -17.4 ps shows two lobes. These observations are compatible with the non-hermitian zero mode ω_2_, In particular, the π/2 phase jump between adjacent cavities predicted for the zero mode is translated into a π phase difference between the two extreme cavities. When *Δt_AC_* = -0.6 ps, e.g. three beams are almost simultaneously pumped and all of them are above lasing threshold. Then the three eigen modes are all formed and the far-field pattern is likely the combination of all the far field pattern of the three eigenstates. When *Δt_AC_* = 17.4 ps, e.g. beam C pumps later than A and B, the blue-detuned mode ω_1_ and ω_2_ dominated the emission spectrum. As the blue most detuned hybrid mode will be more efficiently excited, since its spectral overlap with the pumped cavity resonance increases. The far-field pattern at *Δt_AC_* = 17.4 ps is likely the combination of ω_1_ and ω_2_ mode.

**
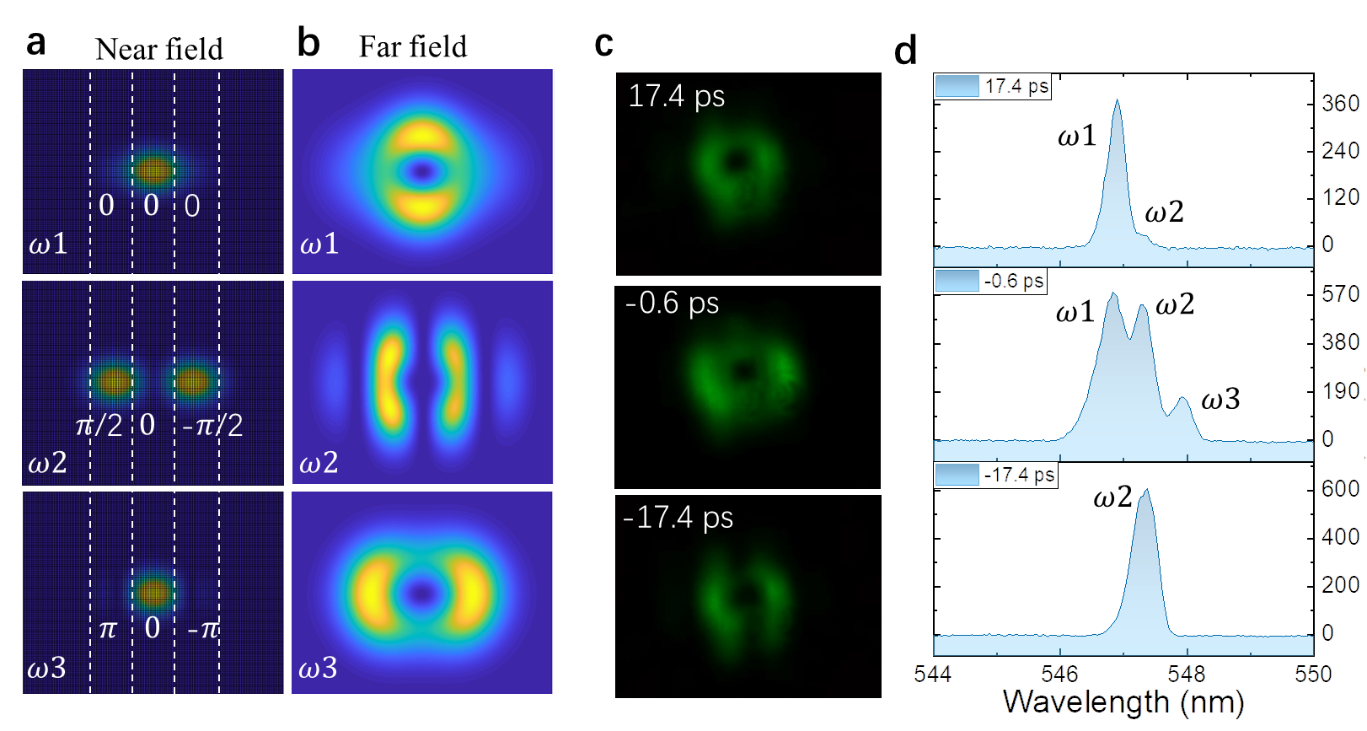
**

**Figure S24.** Observation of the zero mode. Simulated near field **(a)** and far field (**b**) images are displayed for the three eigenstates of the coupled three quasi-BICs with each quasi-BIC cavity oscillating intensity distribution assumed to be a Gaussian function. Vertical white dashed lines in the near field indicate the position of the cavities. From which the ω_2_ corresponding to the zero-mode. (**c**) Far field pattern from different time delay *Δt_AC_*. (**d**) Emission spectrum corresponding to (c).

## Note-7. Distant interactions between multiple quasi-BICs

**Long-range interaction in other directions of the BIC metasurface**

Figure S25 shows the enlarged near field image from the metasurface with pumping power above the threshold. It can be seen that the lasing oscillation of the Γ-BIC mode mainly occurs in the Γ-X direction, and secondarily in the Γ-M direction. Based on this, we measured the emission spectra of the metasurface when pumped by two pump beams with the same separate distance but at different alignment angles, as shown in Fig. S25(c). The insert in Fig. S25(a) exhibit the configuration of two beams pumped on the sample. Here, 0 (90) degrees is defined as the alignment of the two beams parallel to the Γ-X direction, and 45 degrees is defined as the alignment line along the Γ-M direction. In all measurements, the pumping power are set as *P_A_*=1.2 *P_th_*, *P_B_*=0.8 *P_th_*, the distance between two beams is fixed at d = 25 μm.


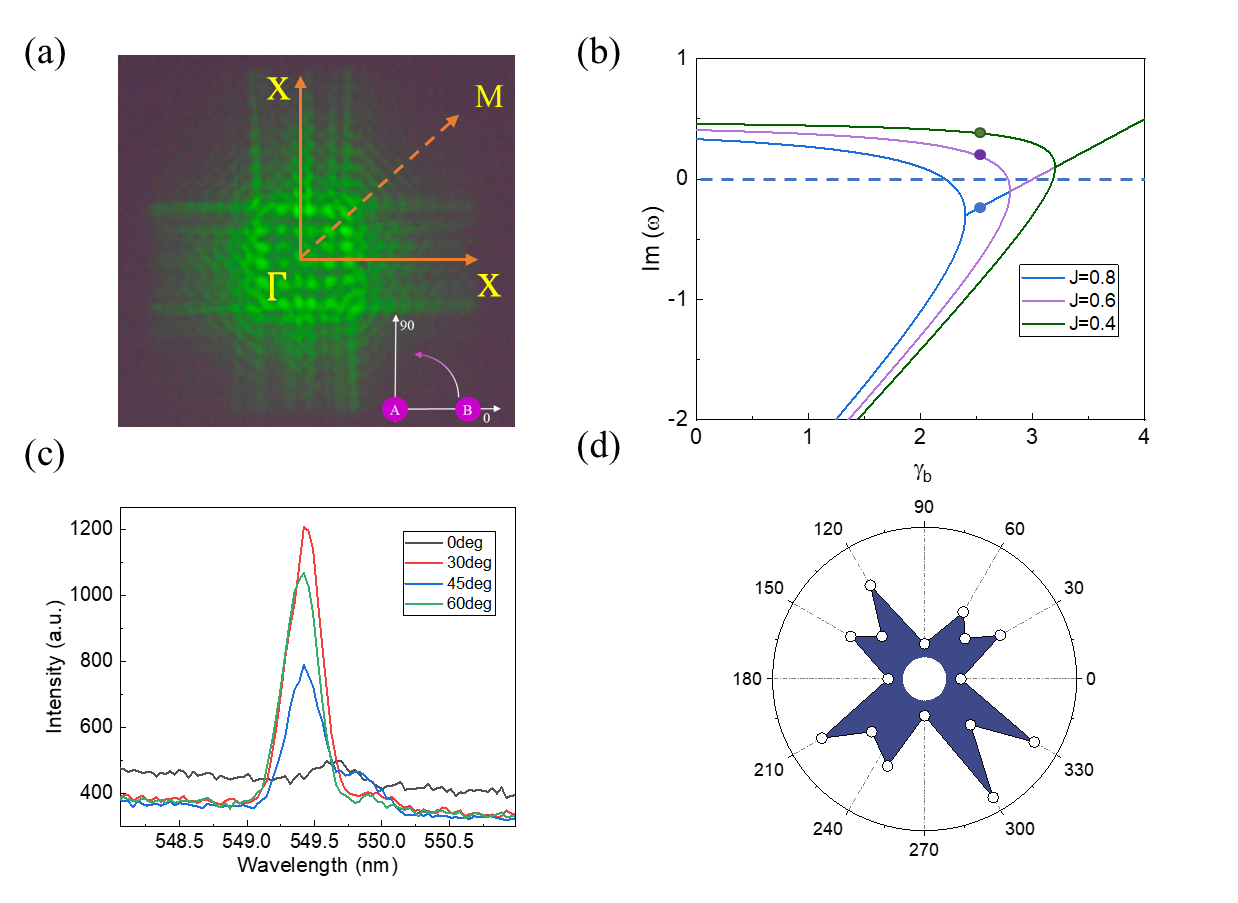


**Figure S25. Long range coupling in other directions.** **(a)** Near field image from the metasurface with pumping power above the threshold. Insert shows the configuration of the two pumping beams, here, 0 degrees is defined as the alignment of the two beams parallel to the Γ-X direction, and 45 degrees is defined as the alignment line along the Γ-M direction. **(b)** Calculated imaginary part of the eigenvalues using the coupled mode theory with different coupling coefficient *J*. (**c**) Emission spectra of the metasurface when pumped by two pump beams with the same separate distance but at different alignment angles. **(d)** Integrated emission intensity of lasing emission when beam B revolves a circle around A as the center.

Figure S25(d) shows the integrated intensity of lasing emission when beam B revolves a circle around A as the center. It can be seen that the lasing emission peak of the sample almost disappears at 0 and 90 degrees, indicate the lasing self-termination. This proves that when the quasi-BICs lines along Γ-X, they can couple each other. While at other angles, such as 30, 45 60 degrees, the sample show obvious lasing emission. However, compared to the situations at 30 degrees and 60 degrees, the lasing emission peak of the sample at 45 degrees decreases again. Using the coupled mode theory, we contribute this to the decrease in the coupling coefficient J. As shown in Fig. S25(b), when γ_b_ is fixed, it can be seen that imaginary part of eigenvalues increase when coupling coefficient decreases. Then blue, purple and green dots in Fig. S25(b) corresponding coupling in 0, 45 and 30 degrees, respectively. Here, the blue dot is located in the lasing self-termination region, while purple and green dots located in single-mode region. However, the imaginary part of green dot is larger, indicating stronger lasing emission.

**Dynamical coupling between multiple quasi-BIC resonators**

To explore the complete dynamics process between multi-body couplings, we place another beam C in the region where ABC form an isosceles triangle, where AC along Γ-M, and AB along Γ-X direction, as shown in the top inset of Fig. S26(c). The distance between A and B is kept in the lasing self-termination region, the time delay is fixed *Δt_AB_* = -16.2 ps. We then measured the evolution of emission spectrum with the time delay between C and A (*Δt_AC_*$= t_{C}-t_{A}$, the negative value means that beam C arrives earlier). The spectrum shown totally different characteristics compared with Fig. 3(b) in the main text, where only A and B pumping the sample and only a single lasing peak in the spectrum when *Δt_AB_* = -16.2 ps. For the three coupled quasi-BICs, when beam C pumping the sample much earlier, *Δt_AC_* = -100.2 ps, for example, the system maintains single mode lasing, as shown in Fig. S26(c). This mainly due to the coupling between A and B are not influenced by C, e.g. the system still in the coupling state between two bodies. As *∆t_AC_* approaching to *Δt_AB_*, e.g, *Δt_AC_* = *Δt_AB_* = -16.2 ps, the single lasing peak gradually decrease, and finally show the characteristic of lasing self-termination. This mainly due to beam C starts to affect the coupling between A and B, and $\gamma_{b}$ increases first as B pumps earlier than A. As *∆t_AC_* further approaches the zero, C begins to affect both A and B, e.g. the system enters the region of three-body coupling region. As shown in the spectrum, when -16.2 ps < *∆t_AC_* < 0 ps, lasing peaks reappear in spectrum. Figure S26(a) and (b) calculated the real and imaginary parts of the eigenvalues using the coupled mode equations upon the three-body coupling. From Fig. S26 (b), it can be seen that when $\gamma_{C}$ is small, the imaginary parts of all three eigenvalues are below the threshold. As $\gamma_{C}$ gradually increases, ω_2_ first exceeds the threshold, and then followed by ω_1_. In other words, the original system is in the lasing self-termination interval. As ∆t_AC_ approaches zero, that is, $\gamma_{C}$ gradually increases, the system will gradually exhibit single-mode or even dual-mode lasing emission, which is in very good agreement with the experimental spectrum, as shown in the inset of Fig. S26(c). When *∆t_AC_* passed the zero point and further increased, the dual-mode lasing peaks gradually decreases, and finally only the single-mode lasing peak retained, meaning the system reverts back to the coupling between A and B.


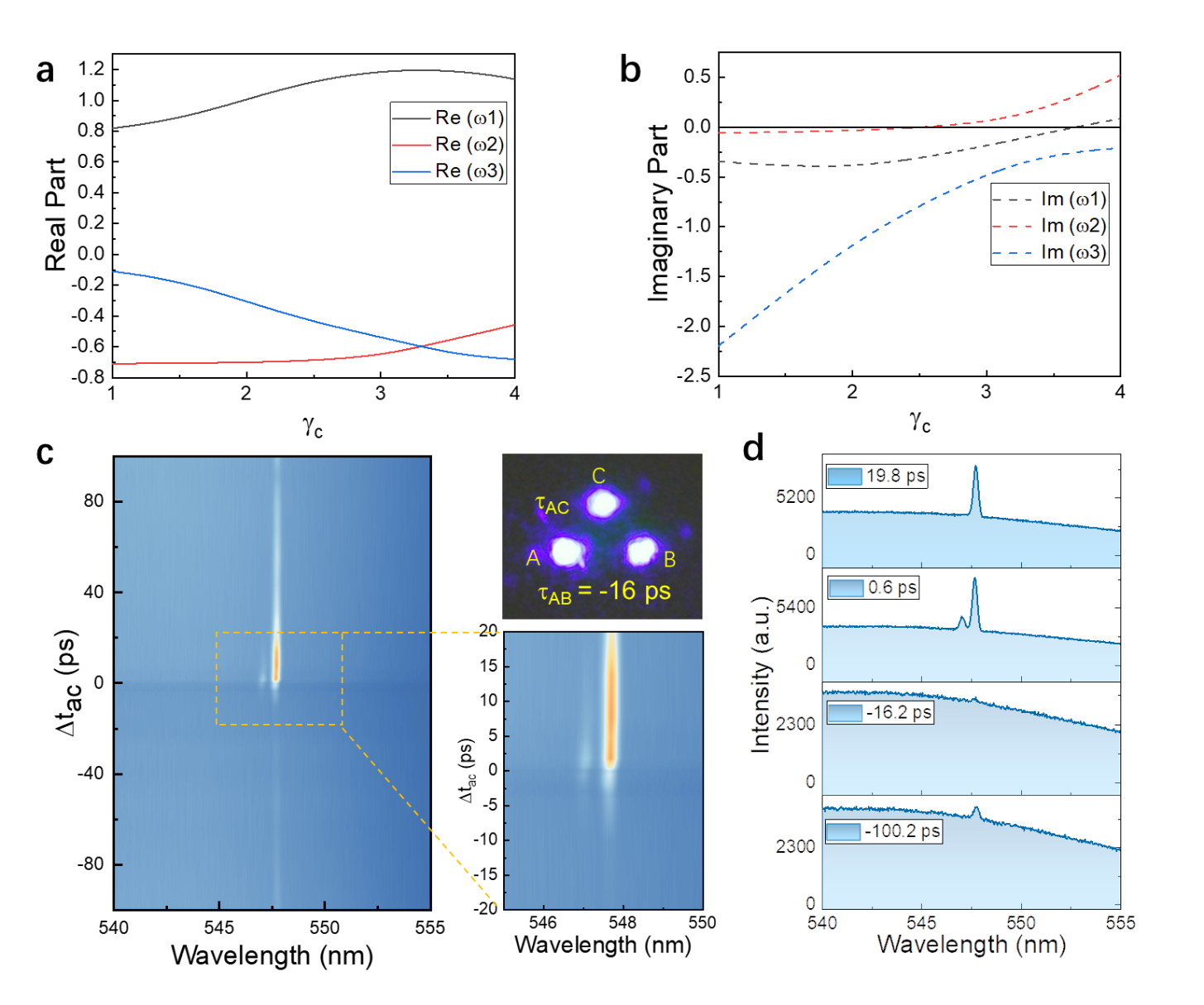


**Figure S26.** Dynamical control of lasing actions of three coupled quasi-BICs. (**a**) and (**b**) show the real and imaginary parts of the eigenvalues of three coupled quasi-BIC resonators as a function of $\gamma_{c}$. For simplicity, we define $\omega_{0}=0$, $\gamma_{a}=4$, $\gamma_{b}=2.6$, $J_{ba}=J_{ab}$=1, $J_{ac}=J_{ca}=J_{bc}=J_{cb}$=0.5, $\kappa_{a}=\kappa_{b}=3.4$. The lasing threshold is defined as *Im(ω)* = 0. **(c).** The evolution of emission spectrum as a function of *∆t_AC_*, with *∆t_AB_* =-16.2 ps. *P_A_*=1.2 P_th_, *P_B_*=0.8 *P_th_*, and *P_C_*=0.8 *P_th_*. Top inset shows the configuration of the three pumping beams, bottom inset shows the detailed spectrum at -20 ps < *∆t_AC_* < 20 ps. (**d**). Emission spectra at particular time delays, i.e., *Δt_AC_* =-100.2 ps, -16.2 ps, 0.6 ps, 19.8 ps, respectively.
